# A systematic review and network meta-analysis of the safety of early interventional treatments in rheumatoid arthritis

**DOI:** 10.1093/rheumatology/keab429

**Published:** 2021-07-19

**Authors:** Maryam A Adas, Victoria B Allen, Mark Yates, Katie Bechman, Benjamin D Clarke, Mark D Russell, Andrew I Rutherford, Andrew P Cope, Sam Norton, James B Galloway

**Affiliations:** Centre for Rheumatic Diseases, King’s College London, London, UK

**Keywords:** early rheumatoid arthritis, DMARD naïve, treatment strategies, adverse events, network meta-analysis

## Abstract

**Objectives:**

To evaluate the safety of treatment strategies in patients with early RA.

**Methods:**

Systematic searches of MEDLINE, EMBASE and PubMed were conducted up to September 2020. Double-blind randomized controlled trials (RCTs) of licensed treatments conducted on completely naïve or MTX-naïve RA patients were included. Long-term extension studies, post-hoc and pooled analyses and RCTs with no comparator arm were excluded. Serious adverse events, serious infections and non-serious adverse events were extracted from all RCTs, and event rates in intervention and comparator arms were compared using meta-analysis and network meta-analysis (NMA).

**Results:**

From an initial search of 3423 studies, 20 were included, involving 9202 patients. From the meta-analysis, the pooled incidence rates per 1000 patient-years for serious adverse events were 69.8 (95% CI: 64.9, 74.8), serious infections 18.9 (95% CI: 16.2, 21.6) and non-serious adverse events 1048.2 (95% CI: 1027.5, 1068.9). NMA showed that serious adverse event rates were higher with biologic monotherapy than with MTX monotherapy, rate ratio 1.39 (95% CI: 1.12, 1.73). Biologic monotherapy rates were higher than those for MTX and steroid therapy, rate ratio 3.22 (95% CI: 1.47, 7.07). Biologic monotherapy had a higher adverse event rate than biologic combination therapy, rate ratio 1.26 (95% CI: 1.02, 1.54). NMA showed no significant difference between strategies with respect to serious infections and non-serious adverse events rates.

**Conclusion:**

The study revealed the different risk profiles for various early RA treatment strategies. Observed differences were overall small, and in contrast to the findings of established RA studies, steroid-based regimens did not emerge as more harmful.

**Rheumatology key messages**

- For the typical early RA patient, few differences exist between treatments strategies in terms of risk profile.
- Steroid-based treatment strategies did not emerge as more harmful in early RA compared to established RA.

## Introduction

RA has a prevalence of 0.5–1.0% [1]. It is characterized by chronic joint inflammation, synovial hyperplasia and systemic manifestations. Without adequate treatment, RA can lead to severe joint deformity and disability [2, 3], impacting upon patients’ quality of life and work ability [4, 5].

The historic approach to treating newly diagnosed RA involved bed rest, analgesia, and (subsequently) DMARDS [6]. The introduction of treat-to-target (T2T) strategies optimized outcomes for patients, with disease activity guiding adjustments in treatment with conventional synthetic DMARDs (csDMARDs), biologic DMARDs (bDMARDs), targeted synthetic DMARDs (tsDMARDs) and CSs [7].

Patients with RA have increased morbidity and mortality relative to that of the general population, and RA associates with multiple comorbidities, including diabetes, infections and cardiovascular diseases [8]. Potential side effects must therefore be taken into consideration when commencing treatments in patients with RA [9].

There is little evidence available that specifically considers the safety of treatments for early RA. Most evidence is derived from people with well-established disease and a substantial burden of comorbidities. In 2021, we now treat RA more aggressively, and the clinical outcomes are much improved. As a consequence, comparing patients withestablished RA from previous decades to contemporary patients diagnosed in recent years lacks face validity. Understanding treatment risks and benefits early in the course of the disease is important to inform choices about the initial treatment strategy. This will not only help patients and clinicians select the right treatment first time, but also provide an evidence base that is generalizable to the current decade.

This systematic review and meta-analysis investigates the safety outcomes of the various treatment strategies used to treat RA in the existing literature. Our objective was to compare the safety profiles of the early RA treatment strategies, including monotherapy, combination therapy and therapy with and without glucocorticoids.

## Methods

### Database and search strategy

A systematic literature search was performed for human studies using MEDLINE, EMBASE and PubMed databases. Disease search terms included RA, inflammatory arthritis, early arthritis; drug search terms included MTX, SSZ, LEF, Adalimumab, Certolizumab pegol, Etanercept, Golimumab, Infliximab, Tofacitinib, Baricitinib, Upadacitinib, Rituximab, Abatacept, Tocilizumab, and Sarilumab. The full search strategy was published online in advance. The initial search was conducted by two investigators (M.A. and V.A.) with verification from a third reviewer (J.G.).

The primary search was undertaken in April 2020, and a final search was performed in September 2020 to identify new studies that could be incorporated in the review. The study was performed in accordance with the preferred reporting system for systematic reviews (PRISMA) [10], and registered with the international prospective register of systematic reviews (PROSPERO registration: CRD42020195766).

### Eligibility criteria

Eligible studies were English language publications of randomized double-blind clinical trials in adult patients with RA commencing DMARD therapy with a controlled comparator arm. The initial protocol specified the following: (1) patients had to be diagnosed with RA based on the 2010 EULAR/ ACR criteria or ACR 1987; (2) patients had to be treatment naïve (although any dose of steroids or NSAIDs were permitted); (3) RCTs had to use currently licensed drugs at licensed doses for RA; (4) RCTs had to include an active comparator arm. After the initial search, a protocol amendment was introduced to define treatment naïve as MTX naïve or referred to those patients who had received MTX for ≤ 4 weeks and had a disease duration of ≤ 2 years.

Studies were excluded if they were open label or single-blinded trials or conducted on patients with unidentified arthritis. Long-term extension, post-hoc and pooled analysis studies, conference abstracts, case reports, letters to the editor, review articles, case–control studies and cohort studies were also excluded.

### Study selection

Records were managed in EndNote (EndNote X9, Australia). Duplicate articles were identified and removed. Two researchers (M.A. and V.A.) independently screened study titles and abstracts in Rayyan QCRI, Qatar, and eligible studies were selected for inclusion. Further data management was conducted in Microsoft Excel. Disagreements over study eligibility were resolved through discussion with the third reviewer (J.G.). Included studies were exported to EndNote X9 for full- text screening.

### Data extraction

Data extraction was performed in Microsoft Excel, and included study source (author and publication date), study registration number, study characteristics (phase, randomization sequence, type of blinding, pooling of analysis, duration, country, and exclusion and inclusion criteria), patient demographics (sex, age, weight, height, BMI, smoking status), disease duration and activity score (DAS28) pre- and post-intervention, study intervention and comparator details (number of patients in each group, dosage and duration), concomitant steroid usage, type of treatment strategy, serious and non-serious adverse event counts, serious and non-serious infectious adverse event counts plus discontinuation due to adverse events and death.

Only studies with published adverse event rates were included. Supplementary Materials, available at *Rheumatology* online were reviewed. Trials registered on clinicaltrials.gov were checked, for reported adverse events. Data were extracted by both primary reviewers (M.A., V.A.). Risk of bias and study quality were assessed at study and outcome level using the Cochrane Risk of Bias 2 (ROB2) Tool [11].

### Statistical analysis

All statistical analyses were conducted using Stata 16 (StataCorp LLC, USA). Patient exposure years were calculated for intervention and comparator groups using sample size and study duration. The primary outcome of interest was serious adverse events rate, defined as any event associated with the use of i.v. antibiotics, hospital admission, discontinuation of the drug, or death. Secondary outcomes included serious infection and non-serious adverse events rates. The incidences of serious and non-serious adverse events, and serious infection were calculated for each study.

Network meta-analysis (NMA) was conducted to allow comparisons across treatment strategies, even where direct comparisons were sparse. NMA models included both completely drug-naïve, and MTX-naïve studies, and a network meta-regression was performed to explore the effect of naïve status on estimates. Surface under cumulative ranking curve (SUCRA) [12] was estimated in order to indicate the likelihood of different treatment strategies being associated with more harm in regard to serious adverse events, serious infections and/or non-serious adverse events.

For sensitivity analyses, a separate meta-analysis was performed across all studies, on trials with completely drug-naïve and MTX-naïve populations. For RCTs with zero events reported in one or both arms, a continuity correction of a fixed value (0.5) was added to each cell to create an event rate to allow odds ratio comparisons of the event rates between studies, as implemented by the Cochrane Handbook for systematic reviews of interventions [13]. For pairwise meta-analyses, multi-arm studies with a shared control arm were divided by two to be included in the comparison.

The rate ratio of serious adverse events in each study arm was calculated in pairwise meta-analysis using the DerSimonian–Laird random-effects method for dichotomous data.

To illustrate the mean effect size and the CIs for individual studies, estimates were graphically displayed in forest plots.

## Results

### Study characteristics

**Fig. 1:**
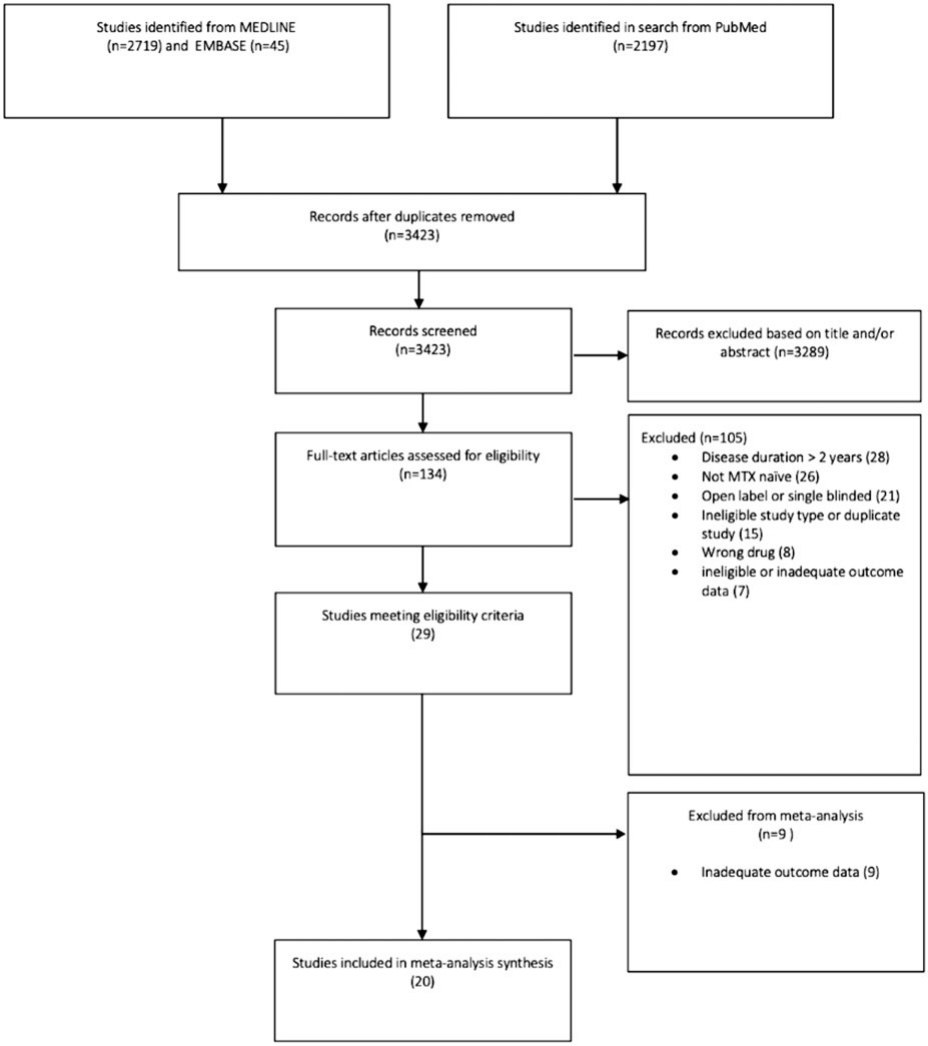
Flow chart of included studies Flow chart of the included studies in the systematic review and network meta-analysis.

The search identified 3423 articles (Fig. 1), of which 29 were eligible RCTs. Nine studies were excluded based on either no adverse event reporting ( three RCTs), inadequate reporting ( four RCTs), or rescue treatment arms precluding analysis according to the original randomized allocation ( two RCTs).

A total of 20 studies, involving 9202 patients, were included in the meta-analysis [14–33]. Articles were published between 1997 and 2020, nine RCTs with 2305 patients conducted on completely drug-naïve patients [14–19, 31–33] and 11 RCTs with 6897 patients on MTX-naïve patients [20–30].

Of the 20 trials, 14 (70%) trials included combination MTX + bDMARD arms [14–27], 1 (5%) included combination MTX + tsDMARD arms [29], 2 (10%) included combination MTX + SSZ arms [31, 32], 2 (10%) included combination MTX + CS arms [18, 33], 4 (20%) included monotherapy bDMARD arms [17, 23–25], 3 (15%) included monotherapy tsDMARD arms [28–30], 2 (10%) included monotherapy SSZ arms [31, 32], and 19 (95%) included monotherapy MTX arms [14–17, 19–33]. Details on all RCTs are shown in Table 1.

**Table 1:** Characteristics of the studies included in the meta-analysis

Author (year), study	Phase of study, country	Population	Intervention	Comparator	Duration of treatment (weeks)	Number of subjects in intervention	Number of subjects in comparator	Age intervention mean (S.D.), years	RA duration intervention mean (S.D.), months	DAS28/ESR intervention mean (S.D.)	
**bDMARD combination and monotherapy**											
**Detert (2013),** **HIT HARD [**14**]**	NR, DE	Treatment naïve	Adalimumab + MTX	MTX	48	87	85	47.2 (12.12)	1.8 (2.09)	6.2 (0.8)	
**Horslev-Petersen (2013),** **OPERA [**15**]**	III, DK	Treatment naïve	Adalimumab + MTX	MTX	52	89	91	56.2	2.8	5.5 (NR)^#^	
**Emery (2016),** **C-EARLY [**16**]**	III, Worldwide	Treatment naïve	Certolizumab pegol + MTX	MTX	52	655	213	50.4 (13.6)	2.9 (4.6)	6.7 (0.9)	
**Bijlsma (2016),** **U-Act-Early [**17**]**	III, NL	Treatment naïve	Tocilizumab Tocilizumab + MTX	MTX	104	103 106	108	55.0 53.0	< 12 ^a^ < 12 ^a^	5.3 (1.1) 5.2 (1.1)	
**Nam (2013), IDEA [** 18 **]**	IV, UK	Treatment naïve	Infliximab + MTX	MTX + i.v. steroid	78^d^	55	57	53.7 (13)	< 12	4.05 (1.04)^Š#^	
**Nam (2014),** **EMPIRE [**19**]**	III, UK	Treatment naïve^e^	Etanercept + MTX	MTX	78	55	55	47.91 (13.58)	6	4.10 (1.14)^#^	
**Atsumi (2016),** **C-OPERA [**20**]**	III, JP	MTX naïve	Certolizumab + MTX	MTX	52	159	157	49.4 (10.3)	4.0 (2.9)	5.4 (1.1)	
**Emery (2008),** **COMET [**21**]**	IV, Worldwide	MTX naïve	Etanercept + MTX	MTX	52	265	263	50.5	8.8 (0.4)	6.5 (1.0) ^c^	
**Tak (2010),** **IMAGE [**22**]**	III, Worldwide	MTX naïve	Rituximab + MTX	MTX	52	249	249	47.9 (13.4)	12	7.1 (1.0)	
**Brumester (2017),** **FUNCTION [**23**]**	III, Worldwide	MTX naïve	Tocilizumab Tocilizumab + MTX	MTX	104	292 290	287	49.9 (13.22) 49 (13.70)	6 6	6.7 (0.99) 6.7 (1.11)	
**Breedveld (2005),** **PREMIER [**24**]**	III, Worldwide	MTX naïve	Adalimumab Adalimumab + MTX	MTX	104	274 268	257	52.1 (13.5) 51.9 (14.0)	8.4 ^b^ 8.4	6.4 (0.9)^c^ 6.3 (0.9)^c^	
**Emery (2015),** **AVERT [**25**]**	III, Worldwide	MTX naïve or MTX (≤10 mg/week) for ≤4 weeks	Abatacept Abatacept + MTX	MTX	52	116 119	116	45.4 (11.9) 46.4 (13.2)	7 6.9	5.5 (1.1)^#^ 5.5 (1.3)^#^	
**Yamanaka (2014),** **HOPEFUL-1 [**26**]**	III, JP	MTX naïve	Adalimumab + MTX	MTX	52^f^	170	163	54.0 (13.2)	3.6	6.6 (0.9)	
**Kavanaugh (2012),** **OPTIMA [**27**]**	IV, Worldwide	MTX naïve	Adalimumab + MTX	MTX	26	515	517	50.7 (14.5)	4.0 (3.6)	6.0 (1.0)^g^	
**tsDMARD combination and monotherapy**											
**Lee (2014),** **ORAL Start [**28**]**	III, Worldwide	MTX naïve	Tofacitinib	MTX	104	770	186	50.3	34.8^h^		6.6
**Fleischmann (2017),** **RA-BEGIN [**29**]**	III, Worldwide	MTX naïve or ≤ 3 weekly doses of MTX	Baricitinib Baricitinib + MTX	MTX	52	159 215	210	51 (13) 49 (14)	22.8 15.6		6.6 (1.1) 6.6 (1.0)
**van Vollenhoven (2020),** **SELECT-EARLY [**30**]**	III, Worldwide	MTX naïve or ≤ 3 weekly doses of MTX	Upadacitinib	MTX	24	317	314	51.9 (12.6)	34.8^i^		5.9 (1.0)^#^
**SSZ combination and monotherapy**											
**HAAGSMA (1997) [** 31 **]**	III/IV, NL	Treatment naïve	SSZ SSZ + MTX	MTX	52	34 36	35	56.8 (13.0) 57 (12.2)	3.1 (1.9) 2.6 (1.4)	4.6 (0.8) 5 (0.8)	
**Dougados (1999) [** 32 **]**	III/IV, Europe	Treatment naïve	SSZ SSZ + MTX	MTX	52	68 68	69	52 (2) 52 (2)	2.9 (0.3) 3.4 (0.3)	4.23^j^ 4.25^j^	
**MTX + steroid**											
**Bakker (2012),** **CAMERA-II [**33**]**	III/IV, NL	Treatment naïve	Prednisone + MTX	MTX	104	117	119	54 (14)	< 12	5.8 (1.3)	

a Reports the symptom duration. ^b^Breedveld (2005) 10–16% of patients’ disease duration up to 3 years. ^c^DAS was not specified. ^d^Nam (2013) only 26 weeks blinded. ^e^Nam (2014) The majority of patients (94%) fulfilled the subsequent 2010 RA classification criteria. ^f^Yamanaka (2014) only 26 weeks blinded. ^g^DAS28 using CRP. ^h^Lee (2014) patients had a mean disease duration of up to 3.4 years. ^i^van Vollenhoven (2020) patients had a mean disease duration of up to 2.9 years. ^j^DAS44. bDMARD: biologic DMARDs; DE: Germany; DK: Denmark; JP: Japan; NL: Netherlands; NR: not reported; tsDMARD: targeted synthetic DMARD.

**Fig. 2:**
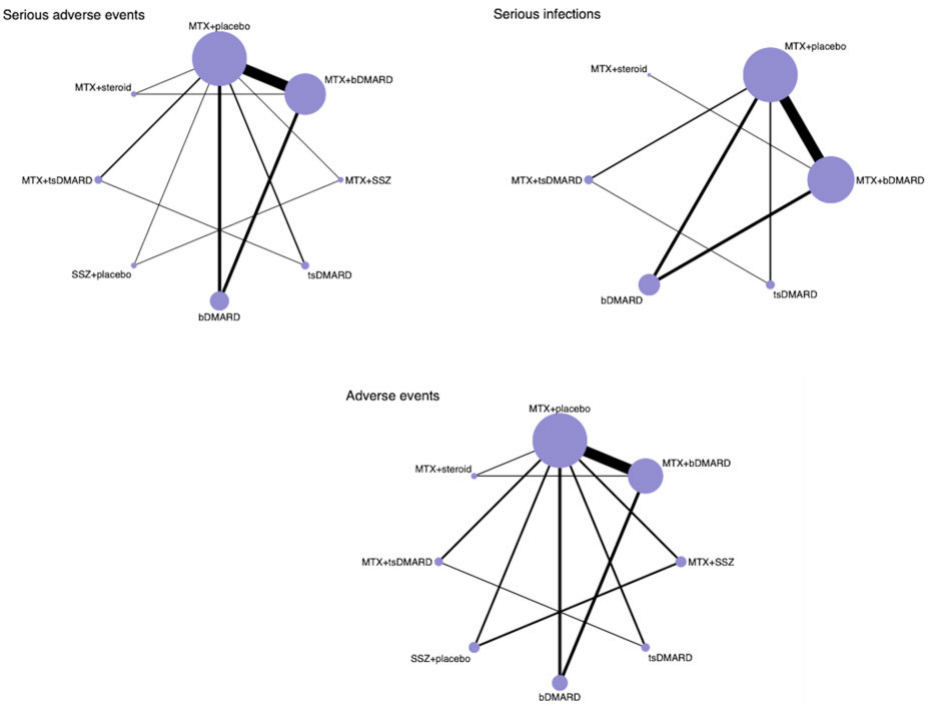
Network plot of comparison of treatment strategies Network plots of comparison of treatment strategies with respect to serious adverse events, serious infections and adverse events (non-serious adverse events) in patients with RA. The size of the circles is proportional to the number of patients in each arm. The line widths are proportional to the number of studies in the comparison. bDMARD: biologic DMARD; tsDMARD: targeted synthetic DMARD.

All 20 studies were included in the NMA. However, the number of studies with data available for each outcome ranged from 17 to 19, as some studies did not report for all outcomes. The networks were built mainly from MTX + placebo studies, and included both completely drug-naïve and MTX-naïve populations (Fig. 2).

### Serious adverse events

Of the 20 studies, 19 were included in the serious adverse events analysis [14–31, 33]. A total of 5572 patients were included in the interventions arms and 3425 patients in the reference MTX monotherapy arms. The pooled incidence rate of serious adverse events per 1000 patient-years was 69.8 (95% CI: 64.9, 74.8).

**Fig. 3:**
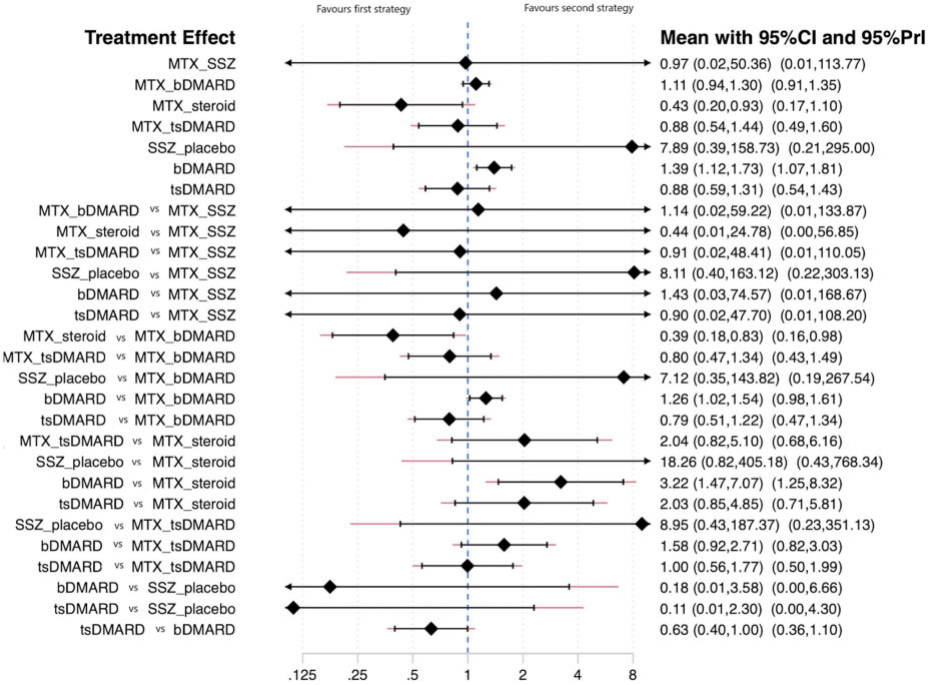
Network meta-analysis of the rate ratio of serious adverse events Network meta-analysis allows indirect comparisons of serious adverse events between treatment strategies. Treatment effects are described comparing the first ( left-hand) strategy with the second ( right-hand) strategy. The first seven strategies were compared with the reference arm, which was MTX + placebo, based on studies with direct comparisons. bDMARD: biologic DMARD; tsDMARD: targeted synthetic DMARD.

There were a number of significant differences between strategies. The pooled rate ratios across both direct and indirect estimates from a model assuming consistency are shown in Fig. 3. Strategies with direct comparisons and pairwise meta-analysis are presented in Table 2. From the comparisons where direct evidence was available, biologic monotherapy showed a higher risk for serious adverse events than MTX monotherapy: rate ratio 1.39 (95% CI: 1.12, 1.73). It was also possible to assess various comparisons between strategies for which direct comparisons were not available. These drew upon small numbers of trials for some analyses. Significant differences emerged favouring MTX + steroid over biologic monotherapy; the serious adverse events rate was 3.22 higher in the biologic monotherapy group (95% CI: 1.47, 7.07). Biologics combination therapy also had higher serious adverse events rates. Biologic monotherapy also had a higher rate ratio of serious adverse events compared with MTX + bDMARD: 1.26 (95% CI: 1.02, 1.54).

**Table 2:** Rates and rate ratios for serious adverse events, serious infections and non-serious adverse events from network meta-analysis and pairwise meta-analysis

	Number of patients	Rate			Rate ratio (PMA)			I2	Rate ratio (NMA)			SUCRA
		Estimate	95% LL	95% UL	Estimate	95% LL	95% UL		Estimate	95% LL	95% UL	
Serious adverse events												
MTX + placebo	3425	71	62.9	80.5	1				1			0.6
MTX + bDMARD	3082	115.2	104.2	126.3	1.07	0.91	1.27	0.0%	1.11	0.94	1.3	0.4
bDMARD	785	139.5	118.9	160.2	1.4	1.05	1.87	0.0%	1.39	1.12	1.73	0.2
tsDMARD	1246	55.6	44.3	66.9	0.93	0.65	1.33	0.0%	0.88	0.59	1.31	0.7
MTX + tsDMARD	215	87.3	56.8	117.8	0.72	0.33	1.57	NA	0.88	0.54	1.44	0.6
SSZ	34	88.2	−11.6	188.1	3.6	0.19	69.75	NA	7.89	0.39	158.73	0.1
MTX + SSZ	36	13.9	−24.6	52.4	0.49	0.01	24.5	NA	0.97	0.02	50.36	0.5
MTX + Steroid	174	11.2	−0.5	22.9	0.41	0.08	2.1	NA	0.43	0.2	0.93	0.9
Overall	**8997**	**69.8**	**64.9**	**74.8**								
Serious infections												
MTX + placebo	3390	18.7	14.2	23.6	1				1			0.5
MTX + bDMARD	3082	26.7	21.4	32.0	0.99	0.70	1.39	0.0%	1.05	0.72	1.51	0.4
bDMARD	785	14.6	7.9	21.3	1.34	0.59	3.06	9.3%	1.22	0.67	2.21	0.3
tsDMARD	1246	13.1	7.7	18.6	1.00	0.51	1.96	0.0%	1.01	0.44	2.28	0.5
MTX + tsDMARD	215	26.8	9.9	43.6	0.53	0.14	1.97	NA	0.74	0.29	1.86	0.7
SSZ	–	–	–	–	–	–	–	–	–	–	–	–
MTX + SSZ	–	–	–	–	–	–	–	–	–	–	–	–
MTX + Steroid	57	12.6	−12.1	37.4	–	–	–	–	0.53	0.04	6.60	0.7
Overall	**8775**	**18.9**	**16.2**	**21.6**								
Non-serious adverse events												
MTX + placebo	3061	937.9	903.3	972.4	1				1			0.5
MTX + bDMARD	2638	1126.8	1088.6	1165.1	0.96	0.92	1.01	2.7%	0.97	0.90	1.05	0.6
bDMARD	511	1142.2	1069.3	1215.2	0.93	0.85	1.01	0.0%	0.96	0.85	1.08	0.7
tsDMARD	1246	1504.4	1445.8	1562.9	1.06	0.98	1.15	0.0%	1.07	0.93	1.21	0.3
MTX + tsDMARD	215	947.1	846.7	1047.5	0.94	0.71	1.23	NA	0.98	0.81	1.20	0.6
SSZ	102	957.4	767.6	1147.3	1.04	0.74	1.47	0.0%	1.04	0.70	1.54	0.4
MTX + SSZ	104	958.5	770.4	1146.7	1.04	0.74	1.47	0.0%	1.05	0.71	1.54	0.4
MTX + Steroid	174	479.1	402.4	555.8	0.93	0.69	1.25	NA	0.97	0.76	1.24	0.6
Overall	**8051**	**1048.2**	**1027.5**	**1068.9**								

Pooled incidence rate per 1000 patient- years and rate ratios for serious adverse events, serious infections and non-serious adverse events across treatment strategies based on studies with direct comparisons. For strategies with zero events reported in one or both arms, a continuity correction of a fixed value (0.5) was added to each cell to create an event rate to allow odds ratio comparisons of the event rates between studies. Higher SUCRA values indicate a greater likelihood of a given treatment causing the least number of events, such that when the SUCRA value is 1, the treatment is the best, and when it is 0, it is the worst. I2 test reflects the the percentage of the variability in effect estimates that is due to heterogeneity. bDMARD: biologic DMARD; I2: I square statistics; NA: not applicable as only one study was available for that strategy; NMA: network meta-analysis; PMA: pairwise meta-analysis; SUCRA: Surface Under the Cumulative RAnking curve; tsDMARD: targeted synthetic DMARD.

Other comparisons did not show any association between the different treatment strategies and the risk of serious adverse events (Fig. 3). The SUCRA approach was used to rank the serious adverse events risk across strategies. SSZ monotherapy was associated with the highest risk of serious adverse events and MTX + steroid with the lowest (Supplementary Fig. S1, available at *Rheumatology* online). No clear inference could be made regarding the risks across the different treatment strategies due to wide CIs, which underlines the uncertainty of the result; this could have been due to the presence of a few direct comparisons in the network; several indirect comparisons were made based on a limited number of studies.

In the sensitivity analysis, the pairwise meta-analysis across all studies ( MTX-naïve and treatment-naïve trials) confirms the results of the NMA for direct comparisons. The pairwise meta-analysis for treatment strategies that had a common MTX arm showed a higher rate ratio of serious adverse events with biologic treatment strategies compared with MTX monotherapy, 1.40 (95% CI: 1.05, 1.87). No other significant differences were observed between other treatment strategies (Supplementary Fig. S2, available at *Rheumatology* online). When limiting the comparison to MTX-naïve trials (Supplementary Fig. S3, available at *Rheumatology* online), the above results were consistent.

However, when limiting the comparison to treatment naïve trials (Supplementary Figs S4–S5, available at *Rheumatology* online), two pairwise meta-analyses were performed, one comparing different treatment strategies with a common MTX monotherapy arm and another comparing different treatment strategies with a common MTX + bDMARD arm. No significant differences emerged between strategies in either analysis.

### Serious infections

The serious infections network included 17 studies [14–30] because 3 studies had to be excluded from the final analysis as they had no reports on serious infections [31–33]. There were 5385 patients in the interventions’ arms and 3390 patients in the MTX monotherapy arms. The pooled incidence rate of serious infections events per 1000 patient-years across all studies was 18.9 (95% CI: 16.2, 21.6).

Studies did not demonstrate a significant difference in risk of serious infections between strategies (Supplementary Fig. S6, available at *Rheumatology* online). Results based on direct comparisons and pairwise meta-analysis are shown in Table 2. A SUCRA graph (Supplementary Fig. S7, available at *Rheumatology* online) and forest plots of the rate ratios for serious infections across all studies (Supplementary Fig. S8, available at *Rheumatology* online) and also limited to treatment status ((Supplementary Figs S9–S11, available at *Rheumatology* online) are presented in the Supplementary Data.

### Non-serious adverse events

The non-serious adverse events network included 17 studies [16–23, 25–33 ] because 3 studies did not comment on the non-serious adverse events and had to be excluded [14, 15, 24]. There were 4990 patients in the interventions arms and 3061 patients in the MTX monotherapy arms. The pooled incidence rate of non-serious infections events per 1000 patient-years across all studies was 1048.2 (95% CI: 1027.5, 1068.9). No significant differences between strategies were observed across studies (Supplementary Fig. S12, available at *Rheumatology* online). Data from direct comparisons and the pairwise meta-analysis are demonstrated in Table 2.

The SUCRA graph (Supplementary Fig. S13, available at *Rheumatology* online) and pairwise meta-analysis results across all studies (Supplementary Fig. S14, available at *Rheumatology* online), when limited to MTX-naïve (Supplementary Fig. S15, available at *Rheumatology* online) and treatment-naïve patients (Supplementary Figs S16 and S17, available at *Rheumatology* online), are presented in the Supplementary Data. When limiting the sensitivity analysis to MTX-naïve trials , the risk of non-serious adverse events was lower with the combination of a bDMARD and MTX, compared with MTX monotherapy, 0.94 (95% CI: 0.89, 0.99).

### Risk of bias

The risk of bias was assessed across different domains according to the Cochrane Collaboration’s Risk of Bias tool [11]. Of the included studies, 14/20 (70%) had an overall low risk of bias. Details on individual study bias assessment can be found in Supplementary Table S1, available at *Rheumatology* online. Trials with recent publication years were least likely to be at high risk of bias across the assessed domains. Older trials were more likely to be assessed as having high risk of bias and excluded from the analysis due to incomplete outcome reporting.

Visual inspection of funnel plots for serious adverse events, serious infections and non-serious adverse events showed no major asymmetry (Supplementary Figs S18–S20, available at *Rheumatology* online). Reporting bias, such as selective outcome reporting and publication bias, could not be ruled out due to large standard errors and low incidence rates (serious infections).

## Discussion

This systematic review and NMA reports on the safety of treatment strategies using licensed drugs in early RA. Differences were seen in the rates of serious adverse events between MTX, bDMARD and steroid treatment. The most clinically relevant and striking finding is that the early CSs use and MTX comes out as a very safe treatment strategy, and were superior in this regard to bDMARD strategies.

To our knowledge, this is the first systematic review and NMA to compare all treatment strategies in early RA. Previous systematic reviews reporting treatment safety in RA mainly focused on certain drug class and/or included patients with long standing disease [34–36].

The NMA showed a statistically significant difference between bDMARD and MTX monotherapy with regards to serious adverse events, with a bDMARD being associated with more harm than MTX treatment. Variations within biologics classes, and substantial study heterogeneity due to differences in patient populations and adverse events reporting, limit the interpretation of the analyses. CIs were wide, with a low number of studies included. Ideally, we would have head- to-head comparisons for all key questions across the treatment strategies in RA, but this is not a feasible expectation. We therefore used NMA to provide insights into comparisons not assessed in clinical trials.

NMA also favoured MTX + steroid and MTX + bDMARD strategies with respect to serious adverse events rates. No differences were apparent when specifically looking into infections and non-serious adverse events. Clinical interpretation of the NMA results and in particular the SUCRA rankings requires extreme caution. Study and population heterogeneity was substantial, and many individual studies were small and provided imprecise estimates of effect size. Crucially, the SUCRA rankings do not provide CIs for the rankings and must be interpreted alongside the tabulated rate ratios.

An a priori belief held by the investigators was that CSs strategies would have a less favourable safety profile, based upon the extensive data in established RA showing the negative impacts of CSs use [37, 38]. Our analyses presented here contradict our prior assumption.

Many national and international guidelines recommend against long term CSs use in RA but advocate the use of steroids in the induction of remission in early disease [39, 40]. The strategy of the use of early CS combined with MTX is much less costly than an early biologic strategy, and alongside our finding of the safety profile advantage that currently recommended treatment strategy for early RA is appropriate.

Results from the direct comparisons using pairwise meta-analysis of completely treatment naïve trials, did not demonstrate a significant difference in serious adverse events risk between MTX + steroid and MTX + bDMARD, rate ratio 0.47 (95% CI: 0.21, 1.02) (Supplementary Fig. S5, available at *Rheumatology* online). However, only one trial was included in this direct comparison [18]. The NMA analysis, on the other hand, included evidence based on indirect comparisons from another study with a steroid arm [33] and multiple studies in the MTX + bDMARD arm [14–27]. The NMA uses information from these indirect comparisons and so provides a different estimate of the effects of MTX + steroid and MTX + bDMARD, rate ratio 0.39 (95%CI: 0.18, 0.83) (Fig. 3). It is important to note that although there is a difference in interpretation based on the *P*-values, the magnitude of the effect is consistent between both methods.

Over time, RCTs reporting guidelines have changed. In 2004, the clinical trials regulations act was set to legislate human trials approval, conduction, monitoring and reporting [41]. In our analyses, steroid data come from historical studies, and that may affect how meaningful the results are. The differences observed in the included studies’ regulation requirements and registration with clinical trial numbers could be another explanation. Of the included trials, three trials had no clinical trials registration number, and two of these were published before 2004. Another five trials did not post their results on clinicaltrials.gov. The sort of magnitude we have seen could be explained by the study bias observed in our included studies.

The explanations for the observed differences may lie in a true effect, or be attributable to variations in study design, sample size, patient selection, and follow-up. Patients enrolled in monotherapy arms are usually patients who failed MTX and MTX combination strategies; thus, these may be selecting patients with high risk. It is relevant to consider the face validity of the findings: the results suggesting a potential benefit of a biologic/MTX combination over biologic monotherapy feel at odds with clinical instinct. However, it is notable that this observation is not novel [42].

The only observed results for serious adverse events overall without differences to serious infections may in part be a reflection of limited study power and the fact that serious infections were rare. It is known that lack of statistical power may affect the interpretation of the analysis [43]. Serious infections were uncommon and did not vary substantially across arms. Of the included trials involving SSZ and steroids strategies, only one commented on serious infections [18]. The observation that we did observe differences for serious adverse events suggests that outcomes other than infection explain the differences. Our study did not explore non-infectious events, and this is an area for future research.

Non-serious adverse events were common across arms, and potentially offer greater power for detecting differences between strategies. In general, the non-serious events were not significantly different across any of the strategies. The lack of any difference indicated by the non-serious adverse events analysis seems surprising, but may reflect the diagnostic heterogeneity of what is coded as non-serious adverse events in a clinical trial. An inference could be that non-serious adverse events are reported during the first year of drug marketing and not considered to be a useful discriminator for pharmacovigilance [44]. It has been published recently that non-serious adverse event collection and reporting in RCTs is inconsistent [45]. Future research in non-serious adverse events may benefit from stratified analysis looking into non-serious adverse events of special interest.

## Strengths and limitations

Treatment of RA has changed across the years, with patients now presenting to clinical practice in the early stages of the disease. A major strength of this work was that we included trials with early RA. An NMA approach was used to compare all treatment strategies in early RA. We limited the analysis to licensed doses of clinical importance, whereas previous publications focused on a single drug class or included all doses [35].

This review had several limitations. Our analysis focused on patients with early RA. It is likely that these patients have fewer comorbidities and less immune dysregulation compared with patients with longstanding disease. This is a possible explanation for the differential response to DMARDs in patients with early RA [46, 47]. The populations recruited, and undoubtedly the selection bias of patients in RCTs, limit the extrapolation of our findings to real world cohorts.

The information on steroids needs to be considered in the context of the dosing regimens: daily oral prednisolone *vs* pulsed methylprednisolone. Bakker *et al.* 2012 [33] used low- dose prednisone of 10 mg per day, with a mean follow- up duration of 109.5 weeks. Nam *et al.* 2013 [18] used i.v. steroids of 250 mg methylprednisolone at week 0, and then placebo at weeks 2, 6, 14 and 22, with a total study duration of 78 weeks. Although the two regimens result in similar steroid burden, there are undoubtedly limitations in our analysis created by combining the studies. Similarly, combining the different classes of biologics into a single group in our analysis may obscure between-group differences. There was heterogeneity within the study populations, treatment-naïve status was not consistent (e.g. regarding prior steroid use), and we were not able to account for comorbidities or geographical differences in our analyses. RCTs usually have insufficient duration and number of patients to detect rare events like serious infectious, so it is possible that the rates in clinical practice are higher.

We originally aimed to explore mortality data. However, many of the included trials did not report either mortality or the cause of death. Of the deaths that were reported, very few were attributable to infectious causes. As we were concerned that there might have been some reporting bias, we did not include this result in our paper.

Finally, it is crucial to acknowledge the limitations of NMA methodology, and that in the absence of direct comparisons within clinical trials, conclusions about comparisons must be made with caution. However, these comparisons are made every day in clinical practice, and network analyses provide the next-best surrogate for head-to-head trials.

In conclusion, for the first time, we have described how different treatment strategies for early RA differ in terms of safety. The message is simple: for the typical early RA patient few differences exist, and for the overwhelming majority of strategies, safety in terms of risks of serious adverse events, serious infections or non-serious adverse event s are not useful discriminators for clinician choice. The most important caveat is that these findings reflect data from clinical trials, which typically exclude patients with significant comorbidity and those patients at extremes of age, limiting the external validity of these results to some extent.

## Supplementary Material

keab429_supplementary_data
