# Supplementary material for: A systematic review and network meta-analysis of the safety of early interventional treatments in rheumatoid arthritis
Source: Rheumatology (Oxford). 2021 Jul 19;60(10):4450–62. doi: 10.1093/rheumatology/keab429 (PMC8487311; doi:10.1093/rheumatology/keab429)
Supplement: keab429_supplementary_data [file keab429_supplementary_data.docx]

Supplementary File

[**Supplementary Table S1. Included studies Risk of Bias assessment** 2](#_Toc68873116)

[**Supplementary Figure S 1. SUCRA graphs for serious adverse events** 4](#_Toc68876019)

[**Supplementary Figure S2. Forest plots for the rate ratio of serious adverse events across all studies** 5](#_Toc68876020)

[**Supplementary Figure S3. Forest plots for the rate ratio of serious adverse events in MTX naïve trials and MTX monotherapy common arm** 6](#_Toc68876021)

[**Supplementary Figure S4. Forest plots for the rate ratio of serious adverse events treatment naïve trials and MTX monotherapy common arm** 7](#_Toc68876022)

[**Supplementary Figure S5. Forest plots for the rate ratio of serious adverse events in treatment naïve trials and MTX+bDMARD common arm** 8](#_Toc68876023)

[**Supplementary Figure S6. Network Meta-analysis plots for the rate ratio of serious infections** 9](#_Toc68876024)

[**Supplementary Figure S7. SUCRA graphs for serious infections** 10](#_Toc68876025)

[**Supplementary Figure S8. Forest plots for the rate ratio of serious infectious across all studies** 11](#_Toc68876026)

[***Supplementary Figure S9. Forest plots for the rate ratio of serious infections in MTX naïve trials and MTX monotherapy common arm*** 12](#_Toc68876027)

[**Supplementary Figure S10. Forest plots for the rate ratio of serious infections in treatment naïve trials and MTX monotherapy arm** 13](#_Toc68876028)

[**Supplementary Figure S11. Forest plots for the rate ratio of serious infections in treatment naïve trials and MTX+bDMARD common arm** 13](#_Toc68876029)

[**Supplementary Figure S12. Network Meta-analysis plots for the rate ratio of non-serious adverse events** 14](#_Toc68876030)

[**Supplementary Figure S13. SUCRA graphs for non-serious adverse events** 15](#_Toc68876031)

[***Supplementary Figure S14. Forest plots for rate ratio of non-serious adverse events across all studies*** 16](#_Toc68876032)

[**Supplementary Figure S15. Forest plots for the rate ratio of non-serious adverse events in MTX naïve trials and MTX monotherapy common arm** 17](#_Toc68876033)

[**Supplementary Figure S16. Forest plots for the rate ratio of non-serious adverse events in treatment naïve trials and MTX monotherapy common arm** 18](#_Toc68876034)

[**Supplementary Figure S17. Forest plots for the rate ratio of non-serious adverse events in treatment naïve trials and MTX +bDMARD common arm** 19](#_Toc68876035)

[**Supplementary Figure S18. Funnel plots of the Network Meta-analysis for serious adverse events** 20](#_Toc68876036)

[**Supplementary Figure S19. Funnel plots of the Network Meta-analysis for serious infections** 20](#_Toc68876037)

[**Supplementary Figure S20. Funnel plots of the Network Meta-analysis for non-serious adverse events** 20](#_Toc68876038)

[References 21](#_Toc68875678)

**Supplementary Table S1. Included studies Risk of Bias assessment**

| Author, study name | Year,  NCT number | Random sequence generation (Selection bias) | Allocation concealment (Selection bias) | Blinding participant& personnel (Performance bias) | Blinding outcome assessment (Detection bias) | Incomplete outcome data (Attrition bias) | Selective reporting (Reporting bias) | Key:  H = high risk of bias L= Low risk of bias U = Unclear risk of bias |
| --- | --- | --- | --- | --- | --- | --- | --- | --- |
| Nam J. (1)  EMPIRE | 2014,  ISRCTN55428162  NR | L | L | L | H | U | H | H |
| Van Vollenhoven (2)    SELECT_  EARLY | 2020,  NCT02706873 | L | L | L | L | L | L | L |
| Detert J. (3)  HIT HARD | 2013,  EudraCT 2006-003146-41  NR | L | L | L | L | U | H | L |
| Nam J. (4)  IDEA | 2013,  ISRCTN48638981  NR | L | L | L | U | U | H | H |
| Lee E. (5)  ORAL Start | 2014,  NCT01039688 | L | L | U | U | L | L | L |
| Horslev-Petersen K. (6)  OPERA | 2013,  NCT00660647  NR | L | L | L | U | U | H | U |
| HAAGSMA C. (7) | 1997,  NA | L | H | U | U | U | L | H |
| Emery P. (8)  C-EARLY | 2016,  NCT01519791 | L | L | L | U | L | L | L |
| DOUGADOS M. (9) | 1999,  NA | L | U | L | U | U | H | H |
| Bijlsma J. (10)  U-Act_Early | 2016,   NCT01034137 | L | L | L | L | L | H | L |
| Bakker M. (11) | 2012,  ISRCTN 70365169  NR | L | L | L | L | U | H | U |
| Atsumi (12)  C-OPERA | 2016,  NCT01451203 | L | L | L | L | L | L | L |
| Emery P. (13)  COMET | 2008,  NCT00195494 | L | L | L | L | L | L | L |
| Tak P. (14)  IMAGE | 2010,  NCT00299104 | U | L | L | L | L | L | L |
| Kavanaugh A. (15)  OPTIMA | 2012,  NCT00420927 | L | L | L | L | L | L | L |
| Burmetster G. (16)  FUNCTION | 2017,  NCT01007435 | L | L | L | L | U | H | L |
| Breedveld F. (17)  PREMIER | 2005,  NA | U | L | L | U | L | H | H |
| Emery P. (18)  Avert | 2015,  NCT01142726 | L | L | U | U | L | L | L |
| Fleischmann R. (19)  RA-BEGIN | 2017,  NCT01711359 | L | L | L | L | L | L | L |
| Yamanaka H. (20)  HOPEFUL-1 | 2014,  NCT00870467 | L | L | L | L | L | L | L |

Table with risk of bias assessment of the included studies using the Cochrane Risk of Bias Tool (Higgins et al. 2011).

L= low risk of bias. U= unclear risk of bias. H= High risk of bias. NCT= National trial number. NA= not applicable. NR= not reported.

**Supplementary Figure S1. SUCRA graphs for serious adverse events**

Figure shows surface under the cumulative ranking curves (SUCRAs); each treatment strategy was ranked based on the estimated probability of being the most effective (causing less events). SUCRA combine the estimated probability from the NMA. Higher SUCRA values indicate a greater likelihood of a given treatment causing the least number of events, such that when the SUCRA value is 1, the treatment is the best, and when it is 0, it is the worst.

**Supplementary Figure S2. Forest plots for the rate ratio of serious adverse events across all studies**


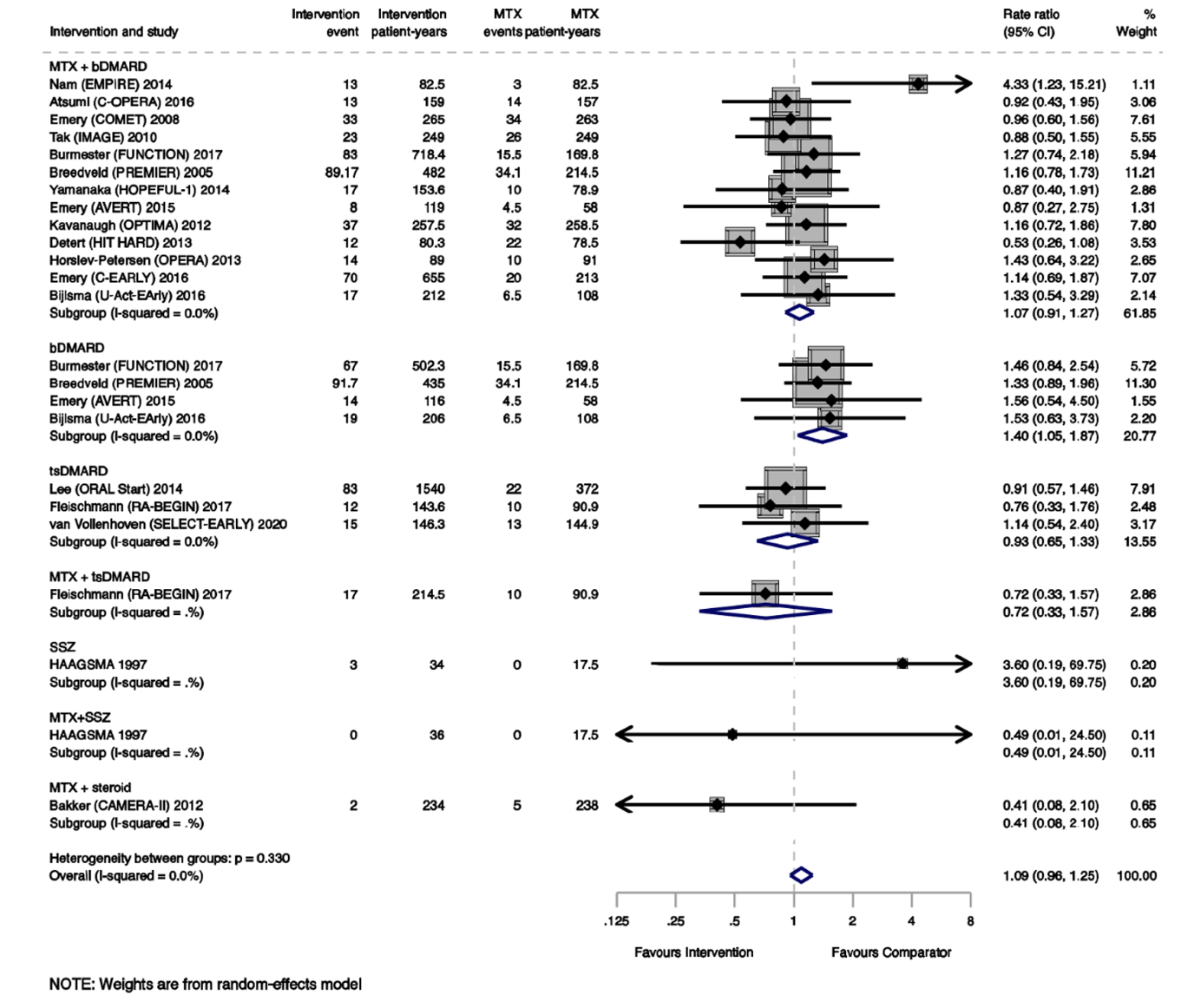


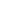


Pairwise meta-analysis forest plots of the rate ratio of serious adverse events of treatment strategies and a common arm (MTX monotherapy). For serious adverse events, 18 studies were included in the analysis (1-3, 5-8, 10-20). Two studies were excluded, one did not report the serious adverse events (9) and the other had no MTX monotherapy arm (4). A total of 5460 patients were in included in the interventions’ arms and 3425 patients in the comparator MTX monotherapy arm.

The pooled rate ratio for serious adverse events comparing different strategies (combination therapy of MTX + bDMARD, MTX + tsDMARD, MTX + SSZ, MTX + steroid, and monotherapy bDMARD, tsDMARD, SSZ) to MTX monotherapy arm was 1.09 (95% CI: 0.96-1.25) with 0% heterogeneity (p=0.33). A higher rate of serious adverse events was seen with biologic treatment strategies than methotrexate monotherapy, 1.40 (95% CI: 1.05-1.87). No significant differences were seen between the other treatment strategies. These findings were consistent when limiting to MTX naïve trials (Supplementary Figure S3). No significant differences across strategies were observed when limiting to treatment naïve trials (Supplementary Figures S4,S5).

bDMARD = biologic disease-modifying antirheumatic drug, MTX = methotrexate, SSZ = sulfasalazine, tsDMARD = targeted synthetic DMARD. Summary diamonds not shown for single studies within group

**Supplementary Figure S3. Forest plots for the rate ratio of serious adverse events in MTX naïve trials and MTX monotherapy common arm**


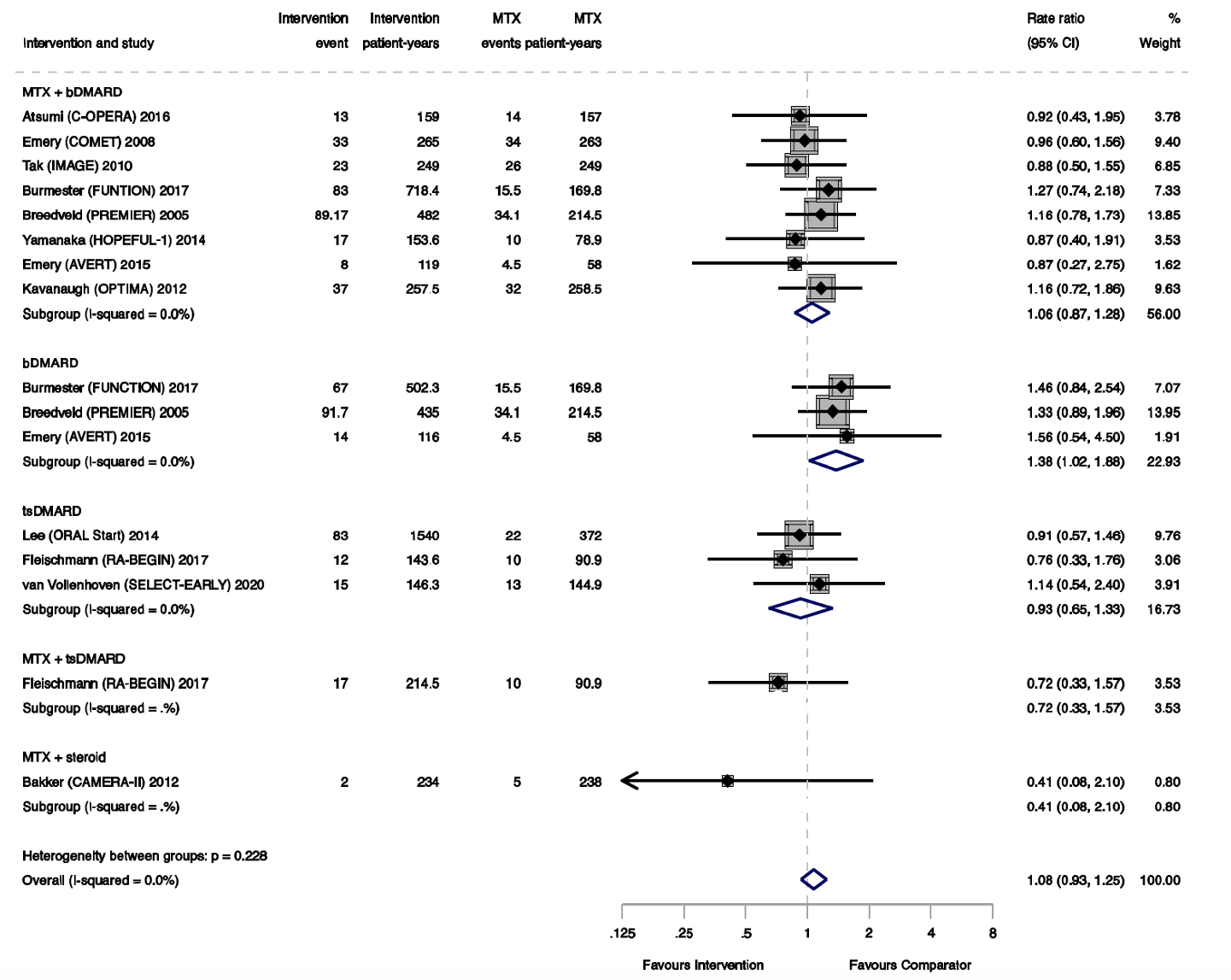


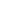


Pairwise meta-analysis forest plots of the rate ratio of serious adverse events of treatment strategies and a common arm (MTX monotherapy) on trials with MTX treatment naïve patients. a higher risk of serious adverse events was found with bDMARD monotherapy compared to MTX monotherapy, 1.38 (95% CI: 1.02-1.88). No significant differences were seen between other strategies.

bDMARD = biologic disease-modifying antirheumatic drug, MTX = methotrexate, SSZ = sulfasalazine, tsDMARD = targeted synthetic DMARD. Summary diamonds not shown for single studies within group.

**Supplementary Figure S4. Forest plots for the rate ratio of serious adverse events treatment naïve trials and MTX monotherapy common arm**


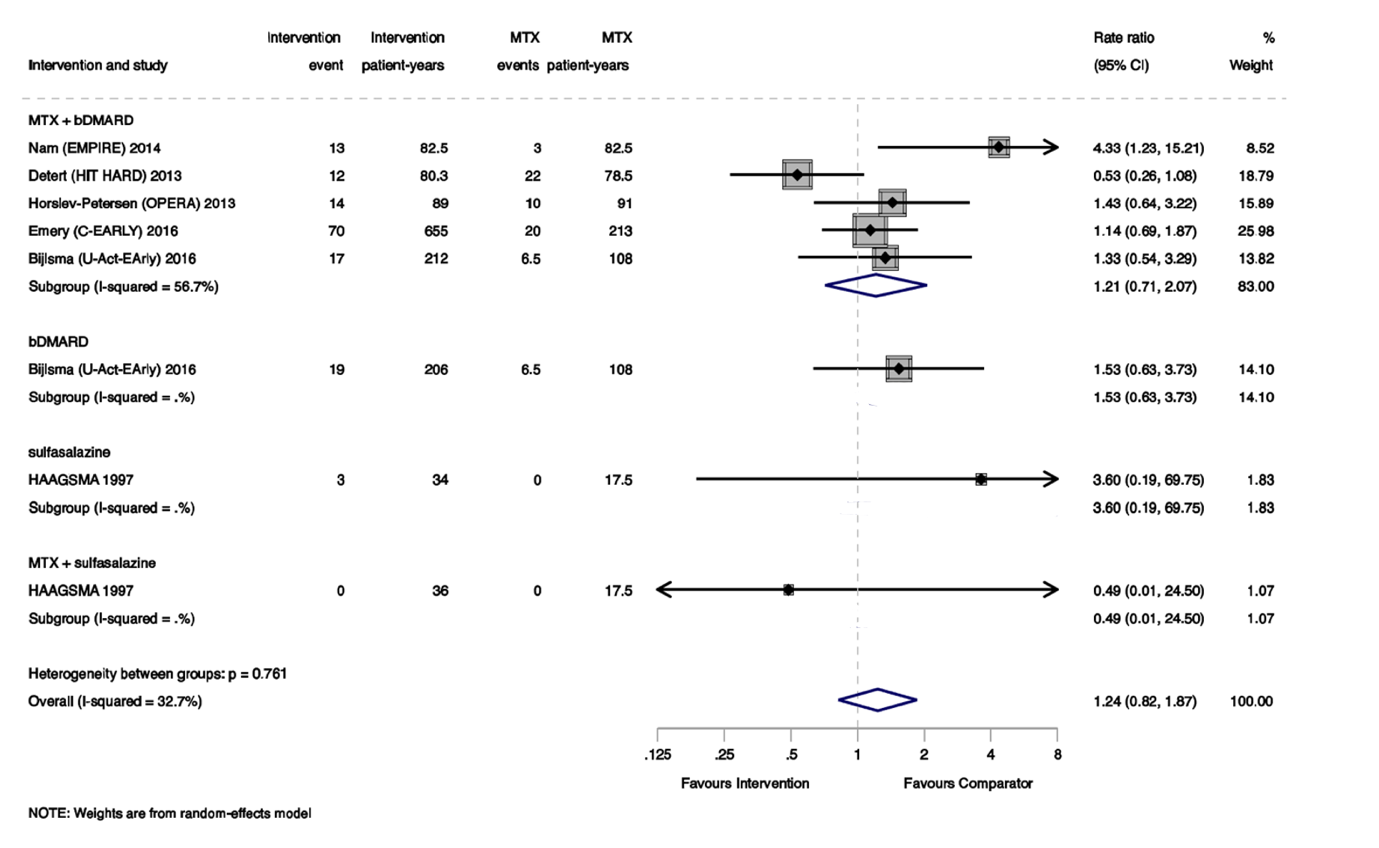


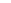

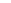

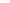

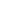


Pairwise meta-analysis forest plots of the rate ratio of serious adverse events of treatment strategies and a common arm (MTX monotherapy) on trials with completely treatment naïve patients. . No significant differences were seen between strategies.

bDMARD = biologic disease-modifying antirheumatic drug, MTX = methotrexate, SSZ = sulfasalazine, tsDMARD = targeted synthetic DMARD. Summary diamonds not shown for single studies within group

**Supplementary Figure S5. Forest plots for the rate ratio of serious adverse events in treatment naïve trials and MTX+bDMARD common arm**


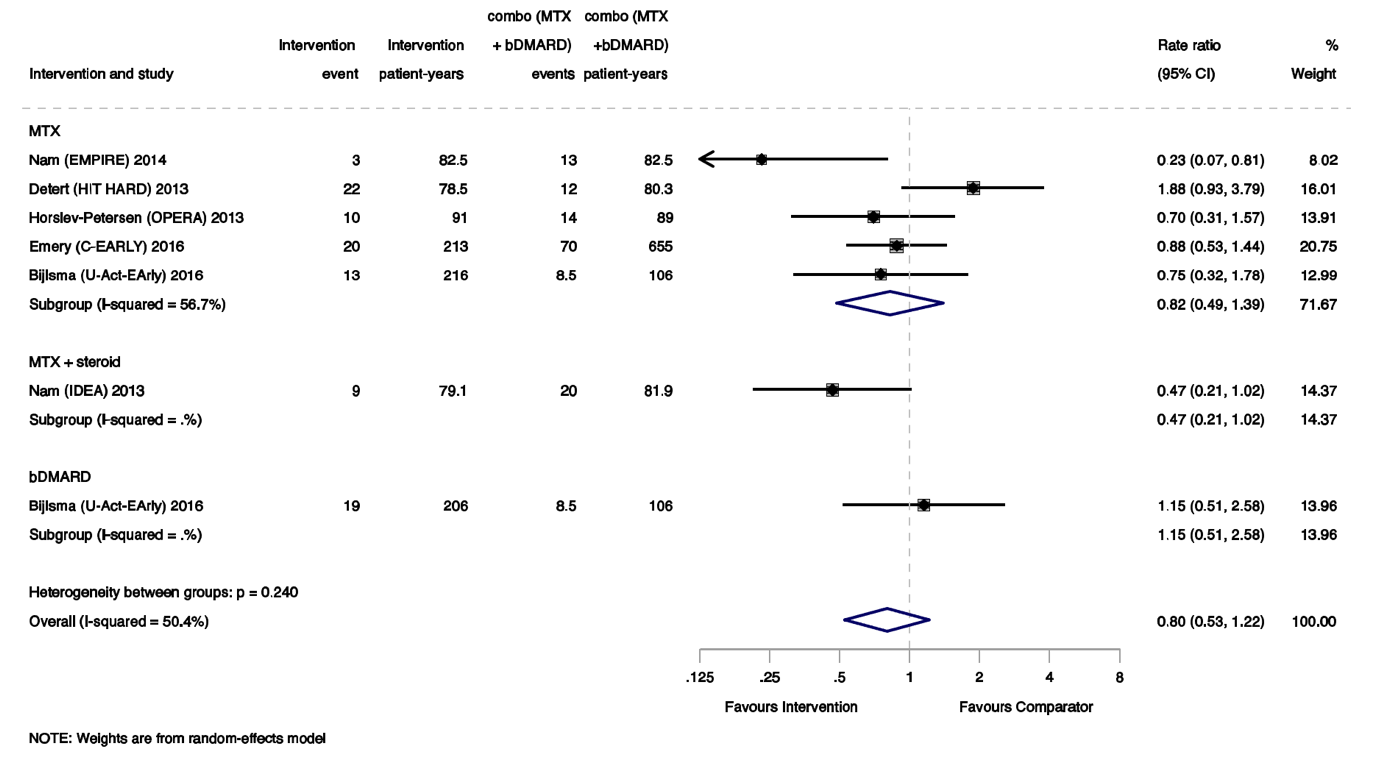


Pairwise meta-analysis forest plots of the rate ratio of serious adverse events of treatment strategies and a common arm (bDMARD + MTX combination therapy) on trials with completely treatment naïve patients. No significant differences were seen between strategies.

bDMARD = biologic disease-modifying antirheumatic drug, MTX = methotrexate, SSZ = sulfasalazine, tsDMARD = targeted synthetic DMARD. Summary diamonds not shown for single studies within group.

**Supplementary Figure S6. Network Meta-analysis plots for the rate ratio of serious infections**


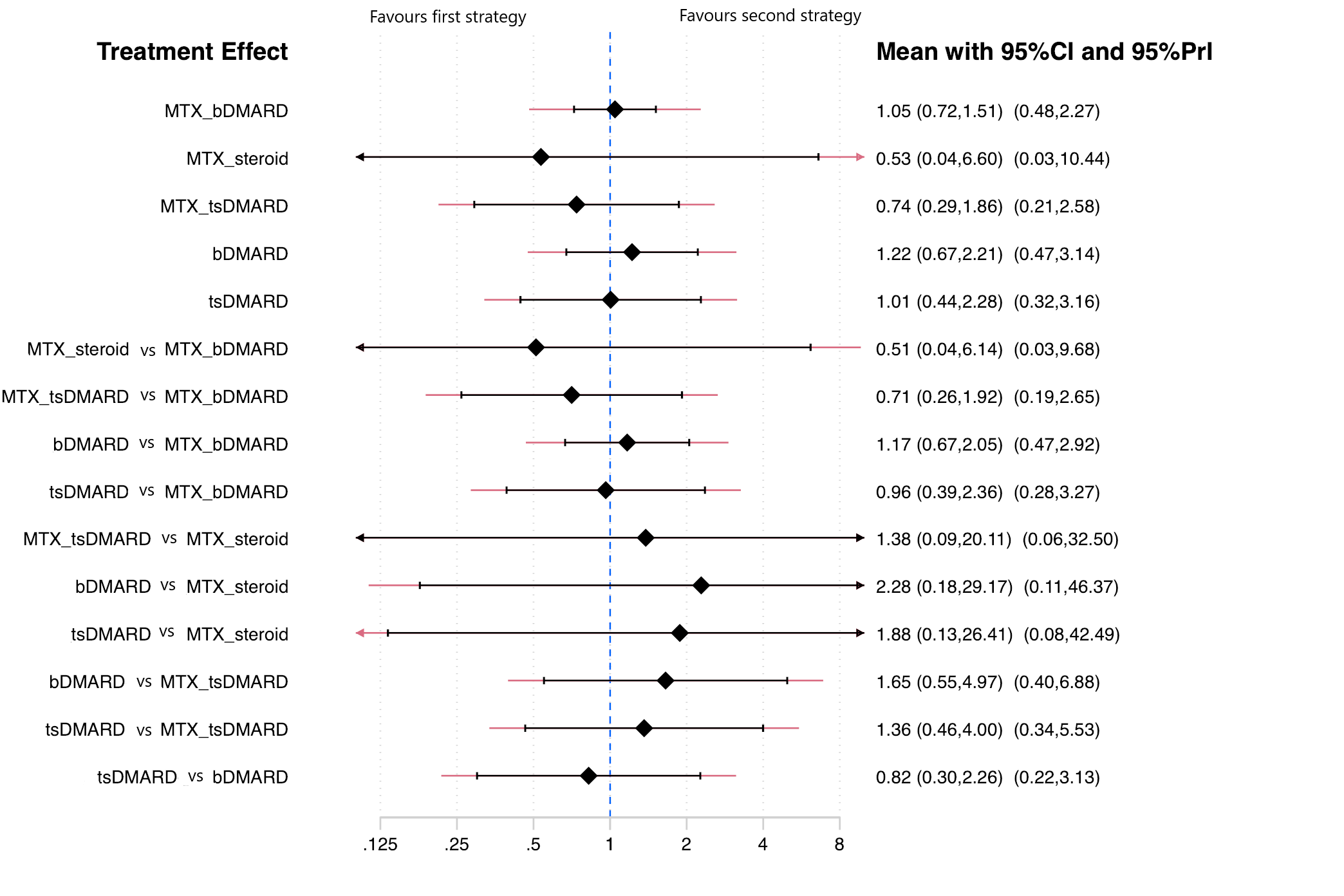


Network meta-analysis allows indirect comparisons for serious infections between treatment strategies. Treatment effects described comparing first (left hand) strategy to second (right hand) strategy. First five strategies are compared to the reference arm, which is MTX+placebo based studies with direct comparisons.

bDMARD = biologic disease-modifying antirheumatic drug, MTX = methotrexate, SSZ = sulfasalazine, tsDMARD = targeted synthetic DMARD. No significant differences were seen between strategies.

**Supplementary Figure S7. SUCRA graphs for serious infections**

Figure shows surface under the cumulative ranking curves (SUCRAs); each treatment strategy was ranked based on the estimated probability of being the most effective (causing less events). SUCRA combine the estimated probability from the NMA. Higher SUCRA values indicate a greater likelihood of a given treatment causing the least number of events, such that when the SUCRA value is 1, the treatment is the best, and when it is 0, it is the worst.

**Supplementary Figure S8. Forest plots for the rate ratio of serious infectious across all studies**


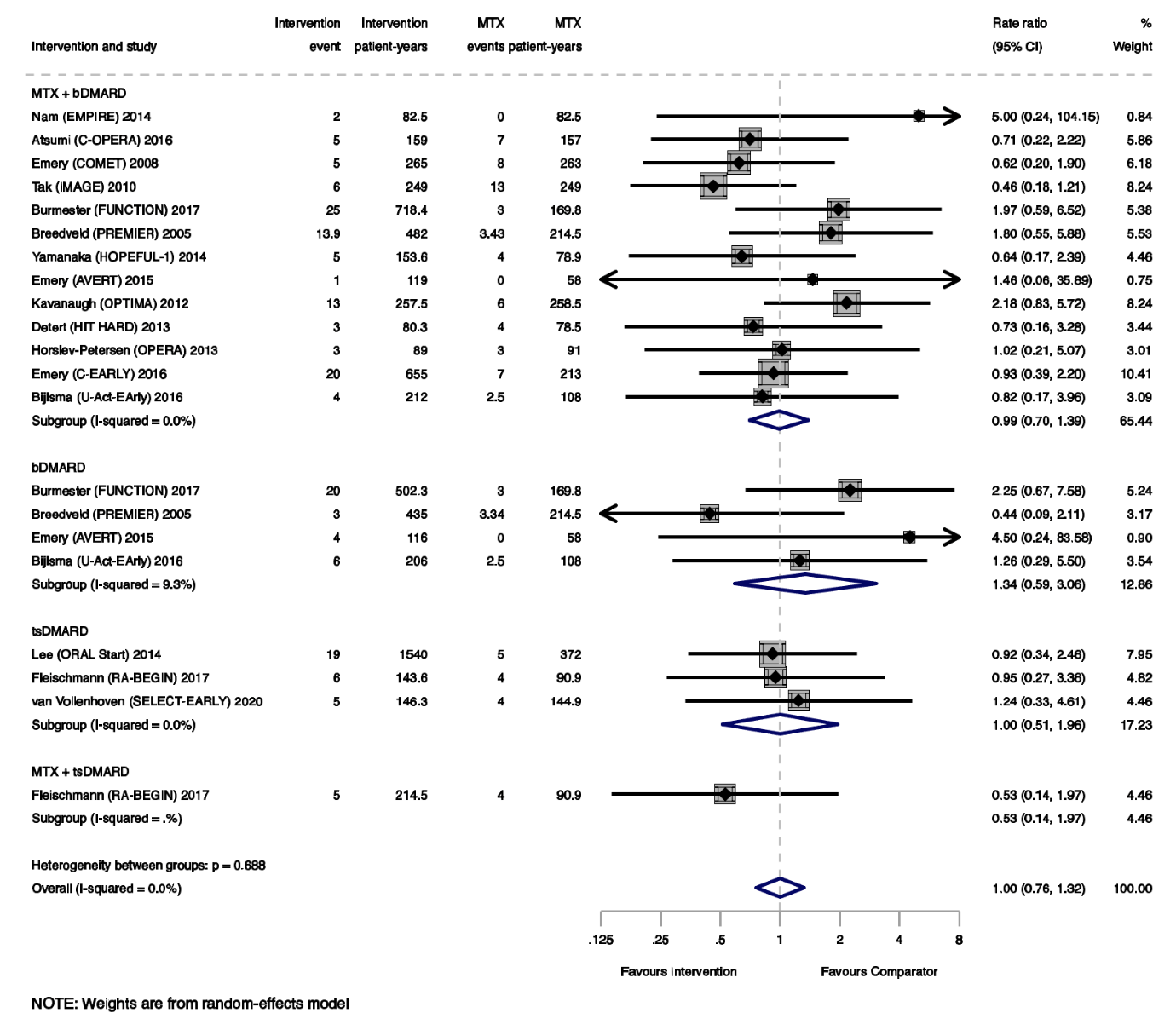


Pairwise meta-analysis forest plots of the rate ratio of serious infections of treatment strategies and a common arm (MTX monotherapy).

16 studies were included in the pairwise analysis (1-3, 5, 6, 8, 10, 12-20). Four studies had to be excluded from the final analysis, three that had no reports on serious infections (7, 9, 11) and one had no MTX monotherapy comparator arm (4). The final analysis included 5273 patients in the interventions’ arms and 3390 patients in the comparator arm (MTX monotherapy).

The pooled rate ratio for serious infections was 1.00 (95% CI: 0.76-1.32) with 0% heterogeneity (p = 0.68) for the following strategies (combination therapy of MTX + bDMARD, MTX + tsDMARD, and monotherapy bDMARD, tsDMARD) excluding mono, combination SSZ and steroid therapy studies since none of these trials reported the serious infection events.

The rate ratios for serious infections did not show any significant imbalance across treatment strategies. The findings were consistent with the overall cohort and when limiting to MTX naïve and treatment naïve trials seen in the sensitivity analysis (Supplementary Figure S9),(Supplementary Figures S10,S11) respectively.

bDMARD = biologic disease-modifying antirheumatic drug, MTX = methotrexate, SSZ = sulfasalazine, tsDMARD = targeted synthetic DMARD. Summary diamonds not shown for single studies within group.

***Supplementary Figure S9. Forest plots for the rate ratio of serious infections in MTX naïve trials and MTX monotherapy common arm***

**
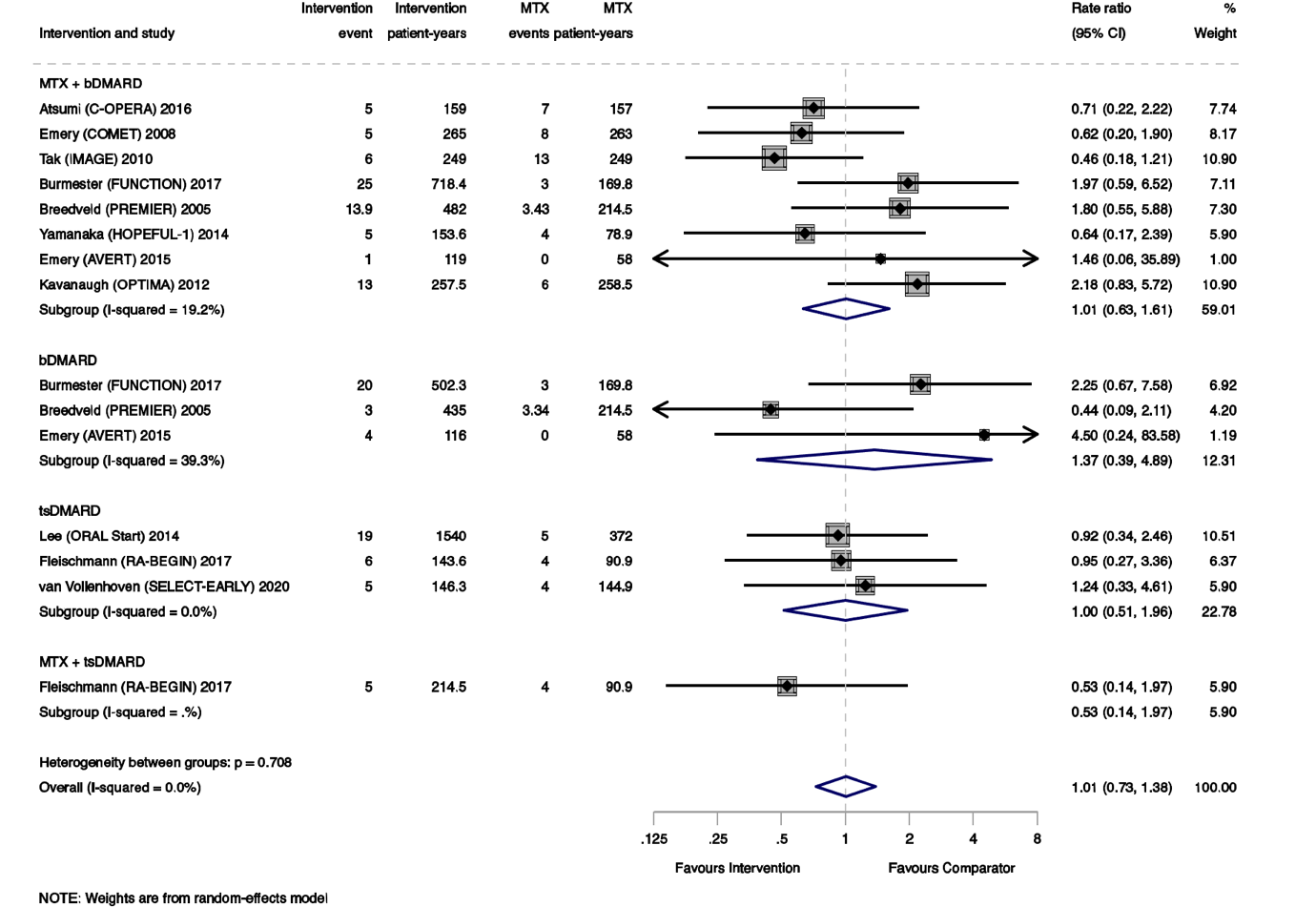
**

Pairwise meta-analysis forest plots of the rate ratio of serious infections of treatment strategies and a common arm (MTX monotherapy) on trials with MTX treatment naïve patients. Serious infection risk was not statistically different between the strategies.

bDMARD = biologic disease modifying antirheumatic drug, MTX = methotrexate, SSZ = sulfasalazine, tsDMARD = targeted synthetic DMARD. Summary diamonds not shown for single studies within group.

**Supplementary Figure S10. Forest plots for the rate ratio of serious infections in treatment naïve trials and MTX monotherapy arm**

**
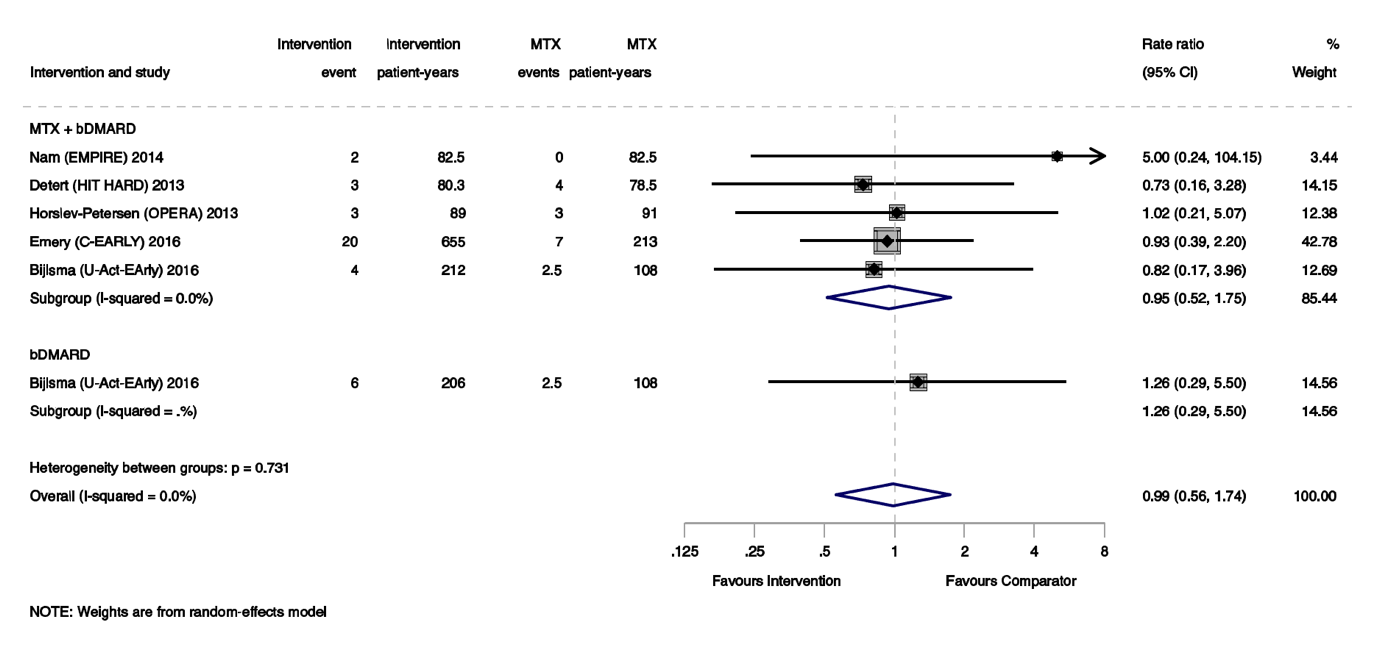
**

Pairwise meta-analysis forest plots of the rate ratio of serious infections of treatment strategies and a common arm (MTX monotherapy) on trials with completely treatment naïve patients.

**Supplementary Figure S11. Forest plots for the rate ratio of serious infections in treatment naïve trials and MTX+bDMARD common arm**



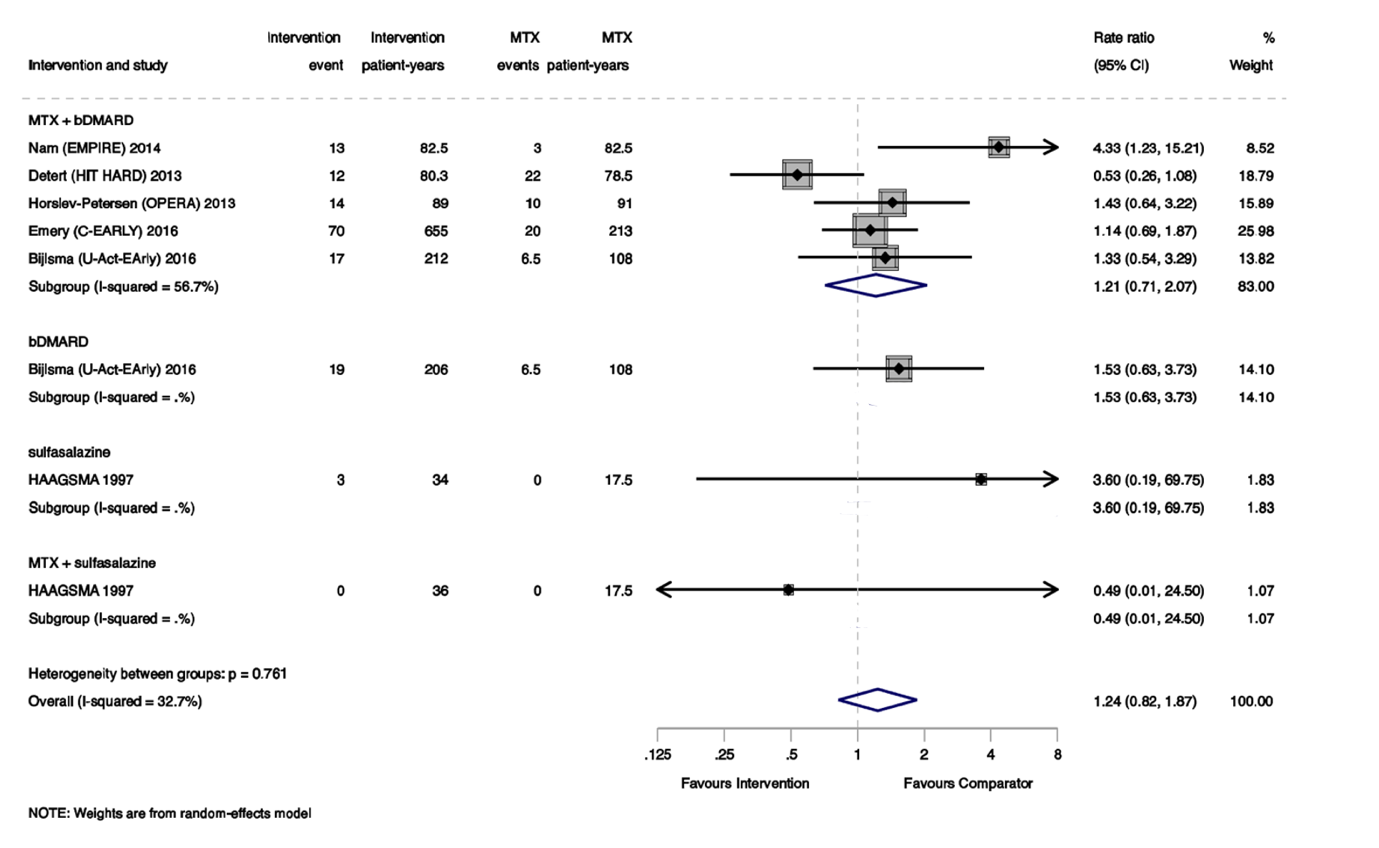


Pairwise meta-analysis forest plots of the rate ratio of serious adverse events of treatment strategies and a common arm (bDMARD + MTX combination therapy) on trials with completely treatment naïve patients. No significant differences were seen between strategies.

bDMARD = biologic disease modifying antirheumatic drug, MTX = methotrexate, SSZ = sulfasalazine, tsDMARD = targeted synthetic DMARD. Summary diamonds not shown for single studies within group.



**Supplementary Figure S12. Network Meta-analysis plots for the rate ratio of non-serious adverse events**


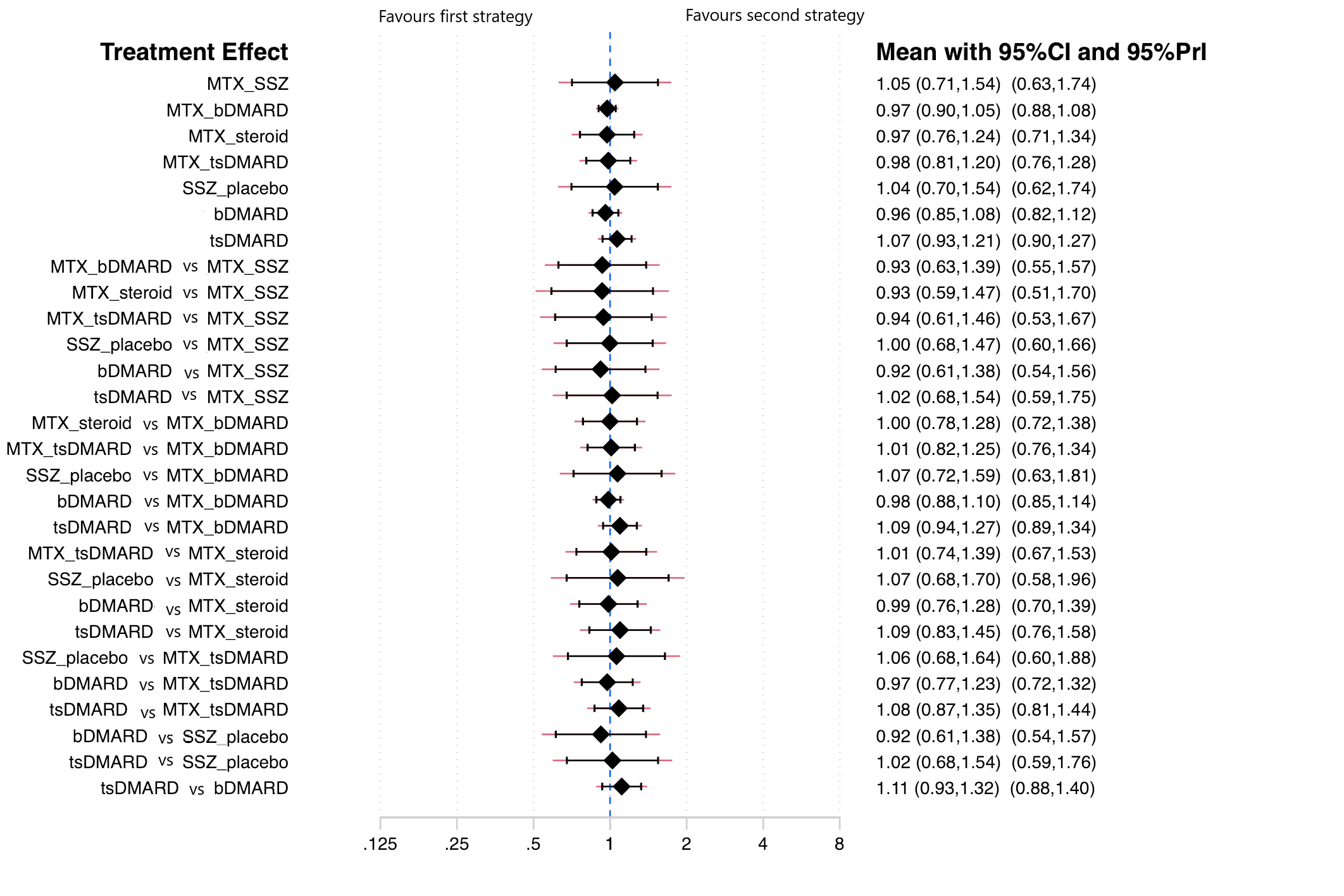


Network meta-analysis allows indirect comparisons for non-serious adverse events between treatment strategies. Treatment effects described comparing first (left hand) strategy to second (right hand) strategy. First seven strategies are compared to the reference arm, which is MTX+placebo based on studies with direct comparisons.

bDMARD = biologic disease-modifying antirheumatic drug, MTX = methotrexate, SSZ = sulfasalazine, tsDMARD = targeted synthetic DMARD. No significant differences were seen between strategies.

**Supplementary Figure S13. SUCRA graphs for non-serious adverse events**

Figure shows surface under the cumulative ranking curves (SUCRAs); each treatment strategy was ranked based on the estimated probability of being the most effective (causing less events). SUCRA combine the estimated probability from the NMA. Higher SUCRA values indicate a greater likelihood of a given treatment causing the least number of events, such that when the SUCRA value is 1, the treatment is the best, and when it is 0, it is the worst.

***Supplementary Figure S14. Forest plots for rate ratio of non-serious adverse events across all studies***


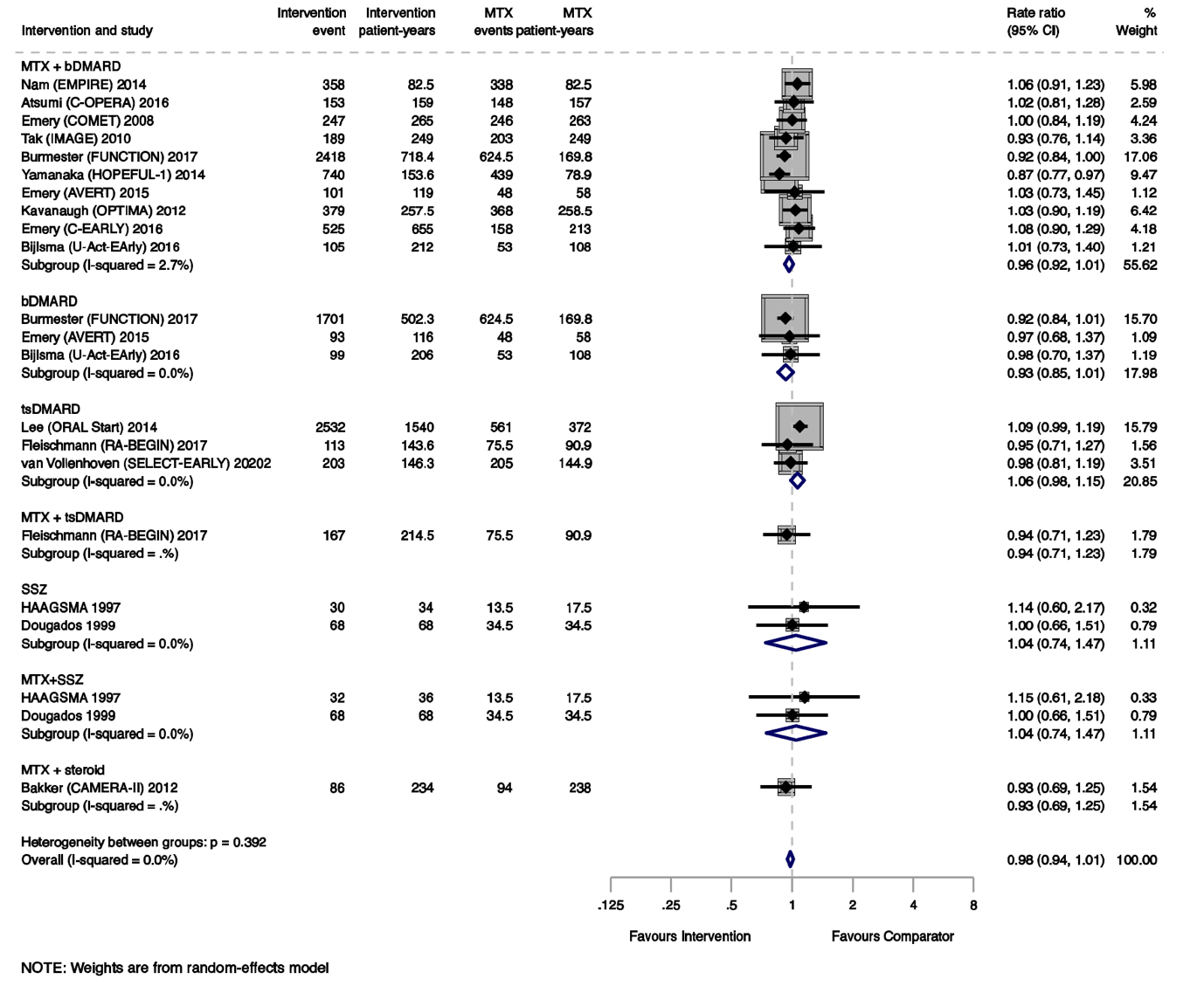


Pairwise meta-analysis forest plots of the rate ratio of non-serious adverse events of treatment strategies and a common arm (MTX monotherapy).

16 studies were included in pairwise meta-analysis (1, 2, 5, 7-16, 18-20). Four studies were excluded, three studies did not comment on the non-serious adverse events (3, 6, 17) and one had no MTX monotherapy arm (4). 4878 patients were included in interventions’ arms and 3061 patients in the MTX monotherapy comparator arm.

The pooled rate ratio for non-serious adverse events for (combination therapy of MTX + bDMARD, MTX + tsDMARD, MTX + SSZ, MTX + steroid, and monotherapy bDMARD, tsDMARD, SSZ) to MTX monotherapy arm was 0.98 (95% CI: 0.94-1.01) with 0% heterogeneity (p = 0.39). No significant differences were seen between strategies.

When limiting to MTX-naïve trials, the risk of non-serious adverse events was lower with combination bDMARD and MTX, compared to MTX monotherapy, 0.94 (95% CI: 0.89-0.99) (Supplementary Figure S15). When limiting to treatment-naïve trials, no significant imbalance were observed (Supplementary Figures S16,S27).

**Supplementary Figure S15. Forest plots for the rate ratio of non-serious adverse events in MTX naïve trials and MTX monotherapy common arm**


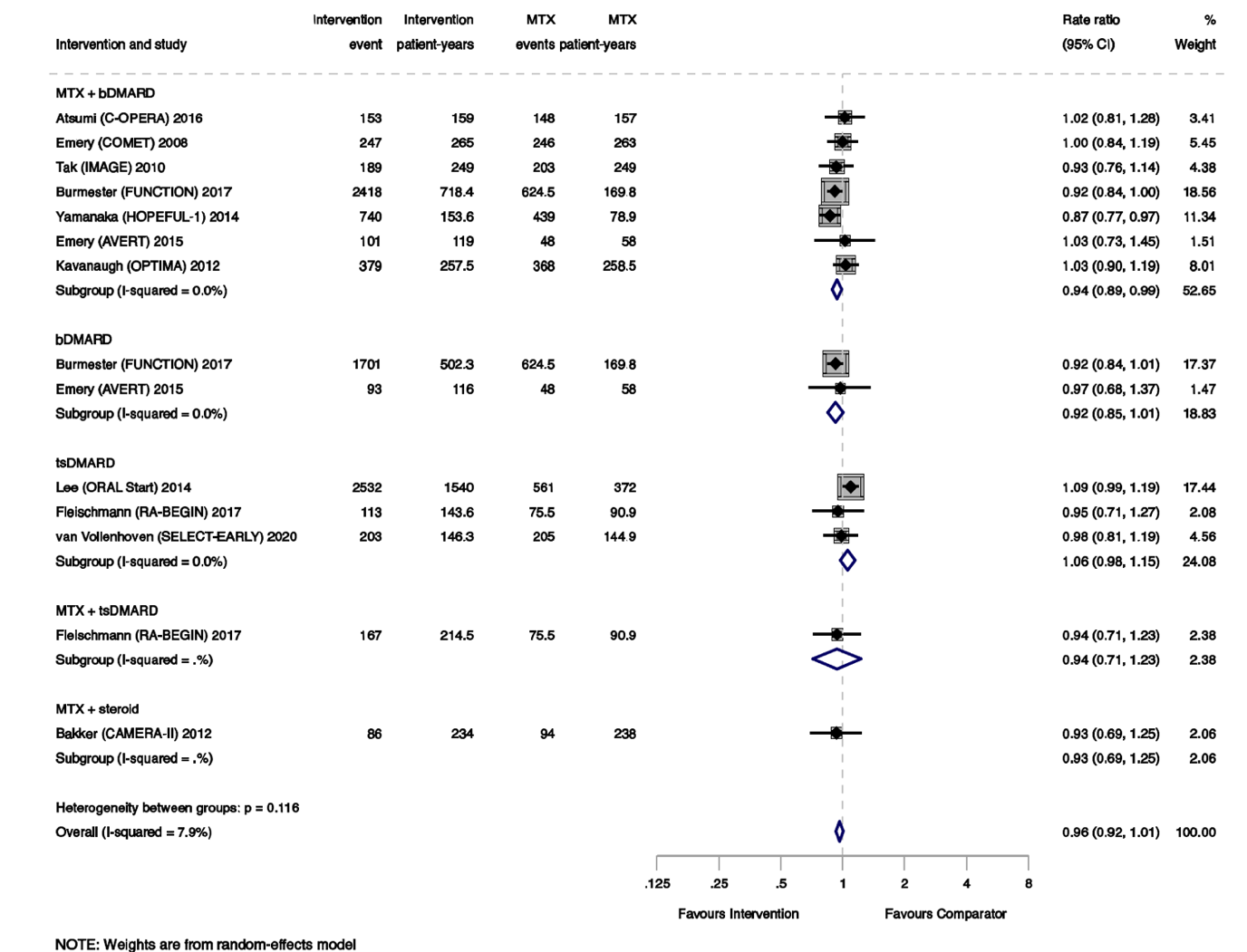


Pairwise meta-analysis forest plots of the rate ratio of non-serious adverse events of treatment strategies and a common arm (MTX monotherapy) on trials with MTX treatment naïve patients. The risk of non-serious adverse events was lower with combination bDMARD and MTX, compared to MTX monotherapy, 0.94 (95% CI: 0.89-0.99). No significant differences were seen between other strategies.

bDMARD = biologic disease-modifying antirheumatic drug, MTX = methotrexate, SSZ = sulfasalazine, tsDMARD = targeted synthetic DMARD. Summary diamonds not shown for single studies within group.

**Supplementary Figure S16. Forest plots for the rate ratio of non-serious adverse events in treatment naïve trials and MTX monotherapy common arm**


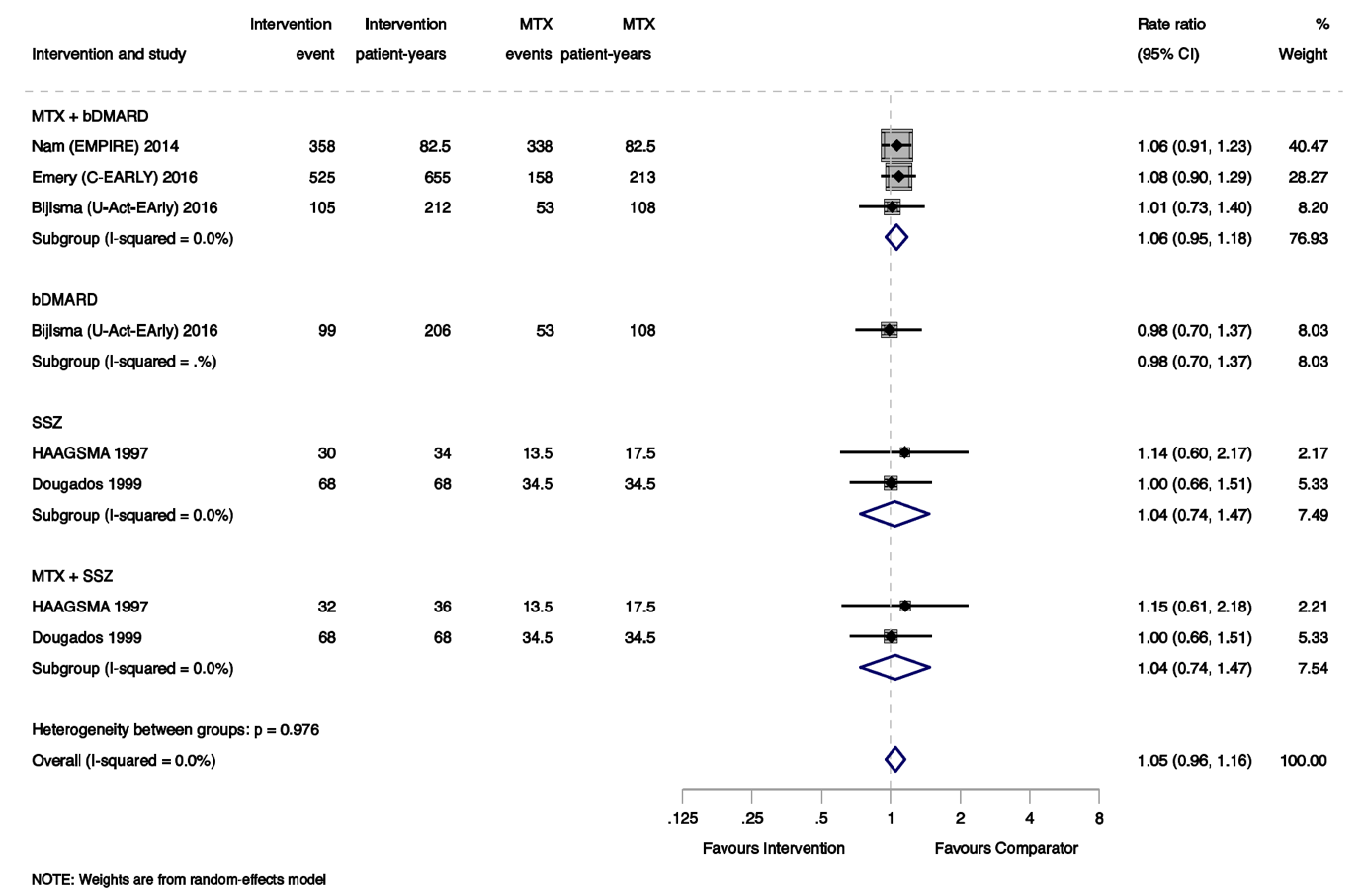


Pairwise meta-analysis forest plots of the rate ratio of non-serious adverse events of treatment strategies and a common arm (MTX monotherapy) on trials with completely treatment naïve patients. No significant differences were seen between strategies.

bDMARD = biologic disease modifying antirheumatic drug, MTX = methotrexate, SSZ = sulfasalazine, tsDMARD = targeted synthetic DMARD. Summary diamonds not shown for single studies within group.

**Supplementary Figure S17. Forest plots for the rate ratio of non-serious adverse events in treatment naïve trials and MTX +bDMARD common arm**


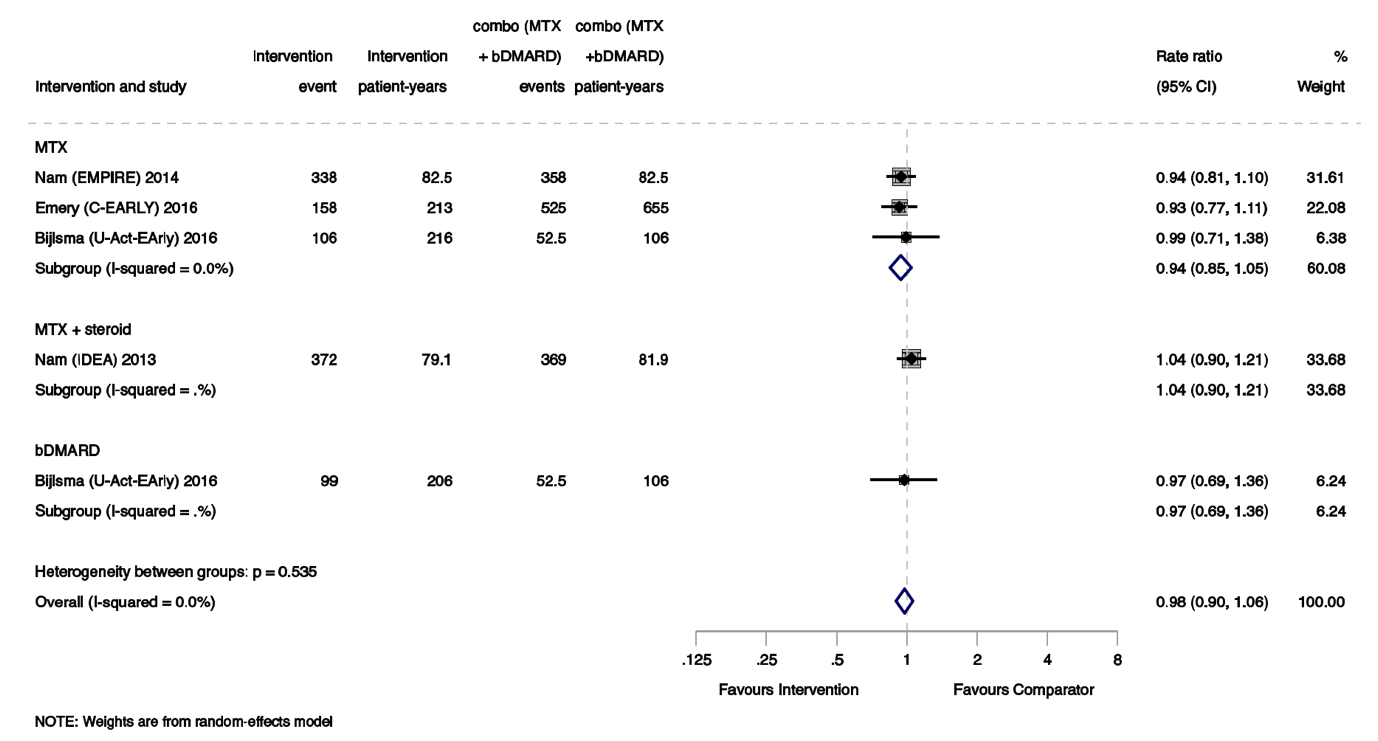


Pairwise meta-analysis forest plots of the rate ratio of non-serious adverse events of treatment strategies and a common arm (bDMARD + MTX combination therapy) on trials with completely treatment naïve patients. No significant differences were seen between strategies.

bDMARD = biologic disease modifying antirheumatic drug, MTX = methotrexate, SSZ = sulfasalazine, tsDMARD = targeted synthetic DMARD. Summary diamonds not shown for single studies within group.



**Supplementary Figure S18. Funnel plots of the Network Meta-analysis for serious adverse events**

**Supplementary Figure S19. Funnel plots of the Network Meta-analysis for serious infections**

**Supplementary Figure S20. Funnel plots of the Network Meta-analysis for non-serious adverse events**

# References

1. Nam J, Villeneuve E, Hensor E, Wakefield R, Conaghan P, Green M, et al. A randomised controlled trial of etanercept and methotrexate to induce remission in early inflammatory arthritis: the EMPIRE trial. Annals of the rheumatic diseases. 2014;73(6):1027-36.

2. van Vollenhoven R, Takeuchi T, Pangan AL, Friedman A, Mohamed MEF, Chen S, et al. Efficacy and Safety of Upadacitinib Monotherapy in Methotrexate‐Naive Patients With Moderately‐to‐Severely Active Rheumatoid Arthritis (SELECT‐EARLY): A Multicenter, Multi‐Country, Randomized, Double‐Blind, Active Comparator–Controlled Trial. Arthritis & Rheumatology. 2020;72(10):1607-20.

3. Detert J, Bastian H, Listing J, Weiß A, Wassenberg S, Liebhaber A, et al. Induction therapy with adalimumab plus methotrexate for 24 weeks followed by methotrexate monotherapy up to week 48 versus methotrexate therapy alone for DMARD-naive patients with early rheumatoid arthritis: HIT HARD, an investigator-initiated study. Ann Rheum Dis. 2013;72(6):844-50.

4. Nam JL, Villeneuve E, Hensor EMA, Conaghan PG, Keen HI, Buch MH, et al. Remission induction comparing infliximab and high-dose intravenous steroid, followed by treat-to-target: a double-blind, randomised, controlled trial in new-onset, treatment-naive, rheumatoid arthritis (the IDEA study). Annals of the Rheumatic Diseases. 2014;73(1):75-85.

5. Lee EB, Fleischmann R, Hall S, Wilkinson B, Bradley JD, Gruben D, et al. Tofacitinib versus methotrexate in rheumatoid arthritis. New England Journal of Medicine. 2014;370(25):2377-86.

6. Hørslev-Petersen K, Hetland ML, Junker P, Pødenphant J, Ellingsen T, Ahlquist P, et al. Adalimumab added to a treat-to-target strategy with methotrexate and intra-articular triamcinolone in early rheumatoid arthritis increased remission rates, function and quality of life. The OPERA Study: an investigator-initiated, randomised, double-blind, parallel-group, placebo-controlled trial. Ann Rheum Dis. 2014;73(4):654-61.

7. Haagsma C, Van Riel P, De Jong A, Van de Putte L. Combination of sulphasalazine and methotrexate versus the single components in early rheumatoid arthritis: a randomized, controlled, double-blind, 52 week clinical trial. British journal of rheumatology. 1997;36(10):1082-8.

8. Emery P, Bingham CO, Burmester GR, Bykerk VP, Furst DE, Mariette X, et al. Certolizumab pegol in combination with dose-optimised methotrexate in DMARD-naïve patients with early, active rheumatoid arthritis with poor prognostic factors: 1-year results from C-EARLY, a randomised, double-blind, placebo-controlled phase III study. Annals of the Rheumatic Diseases. 2017;76(1):96-104.

9. Dougados M, Combe B, Cantagrel A, Goupille P, Olive P, Schattenkirchner M, et al. Combination therapy in early rheumatoid arthritis: a randomised, controlled, double blind 52 week clinical trial of sulphasalazine and methotrexate compared with the single components. Annals of the rheumatic diseases. 1999;58(4):220-5.

10. Bijlsma JW, Welsing PM, Woodworth TG, Middelink LM, Pethö-Schramm A, Bernasconi C, et al. Early rheumatoid arthritis treated with tocilizumab, methotrexate, or their combination (U-Act-Early): a multicentre, randomised, double-blind, double-dummy, strategy trial. The Lancet. 2016;388(10042):343-55.

11. Bakker MF, Jacobs JW, Welsing PM, Verstappen SM, Tekstra J, Ton E, et al. Low-dose prednisone inclusion in a methotrexate-based, tight control strategy for early rheumatoid arthritis: a randomized trial. Annals of internal medicine. 2012;156(5):329-39.

12. Atsumi T, Yamamoto K, Takeuchi T, Yamanaka H, Ishiguro N, Tanaka Y, et al. The first double-blind, randomised, parallel-group certolizumab pegol study in methotrexate-naive early rheumatoid arthritis patients with poor prognostic factors, C-OPERA, shows inhibition of radiographic progression. Annals of the rheumatic diseases. 2016;75(1):75-83.

13. Emery P, Breedveld FC, Hall S, Durez P, Chang DJ, Robertson D, et al. Comparison of methotrexate monotherapy with a combination of methotrexate and etanercept in active, early, moderate to severe rheumatoid arthritis (COMET): a randomised, double-blind, parallel treatment trial. The Lancet. 2008;372(9636):375-82.

14. Tak P, Rigby W, Rubbert-Roth A, Peterfy C, Van Vollenhoven R, Stohl W, et al. Inhibition of joint damage and improved clinical outcomes with rituximab plus methotrexate in early active rheumatoid arthritis: the IMAGE trial. Annals of the rheumatic diseases. 2011;70(1):39-46.

15. Kavanaugh A, Fleischmann RM, Emery P, Kupper H, Redden L, Guerette B, et al. Clinical, functional and radiographic consequences of achieving stable low disease activity and remission with adalimumab plus methotrexate or methotrexate alone in early rheumatoid arthritis: 26-week results from the randomised, controlled OPTIMA study. Annals of the rheumatic diseases. 2013;72(1):64-71.

16. Burmester GR, Rigby WF, Van Vollenhoven RF, Kay J, Rubbert-Roth A, Blanco R, et al. Tocilizumab combination therapy or monotherapy or methotrexate monotherapy in methotrexate-naive patients with early rheumatoid arthritis: 2-year clinical and radiographic results from the randomised, placebo-controlled FUNCTION trial. Annals of the rheumatic diseases. 2017;76(7):1279-84.

17. Breedveld FC, Weisman MH, Kavanaugh AF, Cohen SB, Pavelka K, Vollenhoven Rv, et al. The PREMIER study: a multicenter, randomized, double‐blind clinical trial of combination therapy with adalimumab plus methotrexate versus methotrexate alone or adalimumab alone in patients with early, aggressive rheumatoid arthritis who had not had previous methotrexate treatment. Arthritis & Rheumatism: Official Journal of the American College of Rheumatology. 2006;54(1):26-37.

18. Emery P, Burmester GR, Bykerk VP, Combe BG, Furst DE, Barré E, et al. Evaluating drug-free remission with abatacept in early rheumatoid arthritis: results from the phase 3b, multicentre, randomised, active-controlled AVERT study of 24 months, with a 12-month, double-blind treatment period. Annals of the rheumatic diseases. 2015;74(1):19-26.

19. Fleischmann R, Schiff M, van der Heijde D, Ramos‐Remus C, Spindler A, Stanislav M, et al. Baricitinib, methotrexate, or combination in patients with rheumatoid arthritis and no or limited prior disease‐modifying antirheumatic drug treatment. Arthritis & Rheumatology. 2017;69(3):506-17.

20. Yamanaka H, Ishiguro N, Takeuchi T, Miyasaka N, Mukai M, Matsubara T, et al. Recovery of clinical but not radiographic outcomes by the delayed addition of adalimumab to methotrexate-treated Japanese patients with early rheumatoid arthritis: 52-week results of the HOPEFUL-1 trial. Rheumatology. 2014;53(5):904-13.
